# Safety evaluation of a clinical focused ultrasound system for neuronavigation guided blood-brain barrier opening in non-human primates

**DOI:** 10.1038/s41598-021-94188-3

**Published:** 2021-07-22

**Authors:** Antonios N. Pouliopoulos, Nancy Kwon, Greg Jensen, Anna Meaney, Yusuke Niimi, Mark T. Burgess, Robin Ji, Alicia J. McLuckie, Fabian A. Munoz, Hermes A. S. Kamimura, Andrew F. Teich, Vincent P. Ferrera, Elisa E. Konofagou

**Affiliations:** 1 Department of Biomedical Engineering, Columbia University, New York City, NY 10032 USA; 2 Department of Neuroscience, Columbia University, New York City, NY 10032 USA; 3 Mortimer B. Zuckerman Mind Brain Behavior Institute, Columbia University, New York City, NY 10027 USA; 4 Institute of Comparative Medicine, Columbia University, New York City, NY 10032 USA; 5 Department of Pathology and Cell Biology, Columbia University, New York City, NY 10032 USA; 6 Department of Psychiatry, Columbia University, New York City, NY 10032 USA; 7 Department of Radiology, Columbia University, New York City, NY 10032 USA

**Keywords:** Biomedical engineering, Preclinical research

## Abstract

An emerging approach with potential in improving the treatment of neurodegenerative diseases and brain tumors is the use of focused ultrasound (FUS) to bypass the blood–brain barrier (BBB) in a non-invasive and localized manner. A large body of pre-clinical work has paved the way for the gradual clinical implementation of FUS-induced BBB opening. Even though the safety profile of FUS treatments in rodents has been extensively studied, the histological and behavioral effects of clinically relevant BBB opening in large animals are relatively understudied. Here, we examine the histological and behavioral safety profile following localized BBB opening in non-human primates (NHPs), using a neuronavigation-guided clinical system prototype. We show that FUS treatment triggers a short-lived immune response within the targeted region without exacerbating the touch accuracy or reaction time in visual-motor cognitive tasks. Our experiments were designed using a multiple-case-study approach, in order to maximize the acquired data and support translation of the FUS system into human studies. Four NHPs underwent a single session of FUS-mediated BBB opening in the prefrontal cortex. Two NHPs were treated bilaterally at different pressures, sacrificed on day 2 and 18 post-FUS, respectively, and their brains were histologically processed. In separate experiments, two NHPs that were earlier trained in a behavioral task were exposed to FUS unilaterally, and their performance was tracked for at least 3 weeks after BBB opening. An increased microglia density around blood vessels was detected on day 2, but was resolved by day 18. We also detected signs of enhanced immature neuron presence within areas that underwent BBB opening, compared to regions with an intact BBB, confirming previous rodent studies. Logistic regression analysis showed that the NHP cognitive performance did not deteriorate following BBB opening. These preliminary results demonstrate that neuronavigation-guided FUS with a single-element transducer is a non-invasive method capable of reversibly opening the BBB, without substantial histological or behavioral impact in an animal model closely resembling humans. Future work should confirm the observations of this multiple-case-study work across animals, species and tasks.

## Introduction

Neurodegenerative diseases such as Alzheimer’s and Parkinson’s are characterized by progressive neuronal loss and cognitive decline. Current treatment strategies focus on reducing peptides or proteins that are considered the pathologic hallmarks of these diseases, i.e. amyloid-beta plaques, neurofibrillary tau tangles, and α-synuclein protofibrils or Lewy bodies^1,2^. Despite the reduction of the toxic protein load in the brain, there has been a limited success so far in modifying or reversing the disease^3^. One reason is the inability of existing pharmacological interventions to re-instate the neural network, which has been severely affected due to deficient axonal transport, synaptic loss, and neuronal death^4,5^. Additionally, promising drugs such as antibodies or neurotrophic factors cannot reach the affected brain areas, due to the presence of the blood–brain barrier (BBB)^6^, which is the major limiting factor for drug delivery into the brain^7^. Therefore, potential therapeutic approaches for neurodegenerative diseases should be able to bypass the BBB and, at the same time, reverse cognitive decline through the restoration of depleted neurons.

An emerging physical method to promote drug delivery into the brain is focused ultrasound (FUS) in conjunction with pre-formed circulating microbubbles^8,9^. This modality of FUS-mediated therapy has provided a localized gateway through the intrinsically impermeable BBB for passage of large drugs such as antibodies^10,11^, viral vectors^12–14^, neurotrophic factors^15–17^, proteins^18^, chemotherapeutic agents^19,20^ and nanoparticles^21–23^. Repeated treatments with FUS have been shown safe in multiple animal models, such as rodents^24–27^, canines^28^ and non-human primates (NHPs)^29,30^. Phase I clinical trials of FUS-induced BBB opening in Alzheimer’s disease (AD) patients have been recently completed, without any reported serious side effects^31,32^.

Although FUS-induced BBB opening is primarily intended as a non-invasive drug delivery technique, the temporary disruption of brain homeostasis elicits downstream effects even in the absence of an administered drug. FUS application reduced both the amyloid-beta^24,33–35^ and tau^36^ load through albumin-mediated microglia activation and lysosomal activity^34^ or autophagy^37^, and has been correlated with a restoration of memory in AD mouse models. Short-term proteomic and transcriptomic changes, such as elevation of proinflammatory cytokines, were detected for up to 24 h post-FUS in mice^38^. Such a triggered immune response was also reported to highly depend on the experimental conditions, such as the injected microbubble dose and applied acoustic pressure^39^. The immune response is mild and reversible in rodents^40^, while it correlates with increased angiogenesis within the targeted area^41^. Angiogenesis after BBB opening may also increase cerebral blood flow, improving cognition into late-stage AD in transgenic mice^42^. Apart from promoting vessel growth, moderate immune response expressed as mild activation of microglia, released cytokines and stimulated toll-like receptors has been previously correlated with increased neuronal excitability, neurogenesis, and neurite outgrowth^43^. Furthermore, neurovascular response^44^ and resting-state functional connectivity^45^ in wild-type rodents were affected by localized BBB disruption.

FUS treatments have improved memory in a transgenic mouse model of AD^24,34^. This improvement was correlated with microglia-mediated amyloid plaque^34,35^ or tau protein^36^ reduction, occurring only after BBB opening^46^, and increased neurogenesis within the targeted area^24,47^. Enhanced neuronal formation and plasticity were evident by the increase of doublecortin-positive number of cells and branching in the hippocampal area^24,48^. It has been reported that an increase in BBB permeability is a pre-requisite for neurogenesis stimulation^49^. At the same time, ultrasound exposure itself can elicit neuromodulatory effects in the central nervous system^50–53^. In our previous reports, we studied the cognitive and motor functions of alert NHPs following repeated FUS treatments. We found that there was no negative impact on performance due to the procedure; instead, NHPs had improved accuracy and reaction times while performing a visual task on a touch panel. This was observed during and immediately after FUS-induced BBB opening^30,54,55^. A possible explanation for this short-term change was the neuromodulatory effects detected after FUS-induced BBB opening, evidenced by changes in somatosensory evoked potentials (SSEPs) and blood-oxygen-level-dependent (BOLD) responses^56^. Treatment with high-pressure FUS in rats resulted in SSEP and BOLD changes, which persisted for up to 7 days. Similar BOLD and functional connectivity changes following FUS exposure alone have been reported in NHPs^57,58^, in the absence of BBB opening. Recently, transcranial ultrasound stimulation without microbubbles or BBB opening was shown able to modulate SSEPs in healthy human subjects and also improved memory in patients with probable AD up to 3 months post-exposure^59^.

To date, there has been no demonstration of an immune reaction to FUS treatments in large animal models, such as NHPs, which most closely resemble the human anatomy and brain structure. Cognitive changes following BBB opening have been previously reported in a behaviorally impaired mouse model of AD. Here, we sought to investigate the presence, extent, and resolution of immune response developing in the NHP brain, using immunohistochemistry at two time points after FUS treatment. In separate experiments, we examined the potential impact on the cognitive function of FUS-naïve and wild-type NHPs trained to perform a visual-motor learning test^60,61^. We also assessed the neurological status of treated NHPs on a daily basis, through a standardized neurobehavioral test. We demonstrate that FUS-mediated BBB opening triggers a short-lived immune response within the targeted area of the NHP brain and does not impair performance in a behavioral task immediately after treatment. All FUS procedures were performed using a portable FUS system^62^, including real-time acoustic monitoring, and were conducted with FDA-approved and clinically relevant parameters to ensure translatability of the observations into the clinical setting^31,63^. The overall aim of this multiple-case-study work was to evaluate the histological and behavioral safety profile following a single procedure of BBB opening with a clinical system prototype in a primate model.

## Materials and methods

### Experimental outline

We performed BBB opening in 4 NHPs (rhesus macaques, age: 7.5 ± 0.5 y) to assess both the short-term and long-term effects of ultrasound treatment; 2 for imaging and histology and 2 for imaging and behavioral testing (Table 1). All animal experiments were approved by the Institutional Animal Care and Use Committee (IACUC) of Columbia University and were carried out in accordance with relevant guidelines and regulations. Additionally, the study was in compliance with the ARRIVE guidelines. The experimental plan was produced in coordination with the Center for Devices and Radiological Health (CDRH) of the U.S Food and Drug Administration (FDA) and the veterinary staff from the Institute of Comparative Medicine at Columbia University. Given the imaging, histological and behavioral analysis at both the short-term (i.e., 2 days post-FUS) and long-term (i.e., 18 days post-FUS) time points, which was required by the FDA, this experimental design sought to maximize the amount of information taking into account the number of available NHPs (n = 4) and the available resources. The experiments were thus designed and conducted as multiple case studies, in order to mimic the prospective clinical trial with AD patients (NCT04118764) which involves a single FUS treatment in FUS-naïve subjects. For the context of this study, short-term and long-term effects of FUS refer to effects observed 2 and 18 days post-FUS, respectively. The targeted structure was the prefrontal cortex (PFC). NHP 1 and NHP 2 were sonicated bilaterally in one session, at MI of 0.4 (left PFC) and MI of 0.8 (right PFC), and survived for 2 and 18 days, respectively. Given the focal volume of the transducer (6 × 6 × 49 mm^3^), we anticipated BBB opening in regions outside the PFC. The mechanical index (MI) is defined as the ratio of the peak-negative acoustic pressure in MPa by the square root of the center frequency in MHz, i.e. $MI = P({\text{MPa}}){/}\sqrt {f_{0} ({\text{MHz}})}$. FUS parameters such as MI and acoustic intensity were selected based on previous work and were within the FDA-approved limits for contrast-enhanced ultrasound imaging. Therapeutic pulses were emitted at a center frequency of 0.25 MHz, peak-negative pressure of either 200 kPa (i.e., MI of 0.4) or 400 kPa (i.e., MI of 0.8), pulse length of 10 ms, pulse repetition frequency of 2 Hz, and total treatment time of 2 min. The two pressure amplitudes tested here were selected as the minimum pressure required for BBB opening^64^ and the maximum pressure approved by the FDA for use with circulating Definity microbubbles in imaging applications. Here, we will refer interchangeably to the low MI (i.e., 0.4) treatment as “stable” microbubble activity, and the high MI (i.e., 0.8) as “unstable” microbubble activity. Stable activity is characterized by sustained volumetric microbubble oscillations at the driving frequency and its harmonics. In contrast, unstable activity is characterized by asymmetric expansion and inertia-dominated collapse, resulting in microbubble destruction and jet formation. NHPs 1 and 2 were euthanized through transcardial perfusion, and their brains were extracted and processed for histology. NHPs 3 and 4 had been earlier trained for the behavioral test^60^ and were treated unilaterally targeting the left PFC at MI of 0.4 and 0.8, respectively. Behavioral tests were conducted daily for at least 3 consecutive weeks before and after treatment to assess potential short-term and long-term behavioral changes. NHPs 3 and 4 had no follow-up MRI to avoid bias in the behavioral task performance due to starvation and anesthesia effects.

**Table 1:** Experimental timeline for assessing effects of FUS-induced blood–brain barrier in non-human primates.

Histology/Imaging					Behavioral/Imaging			
Procedure	NHP 1–2-day		NHP 2–18-day		Procedure		NHP 3	NHP 4
FUS treatment	Day 0		Day 0		FUS treatment		Day 0	Day 0
Targets	Left PFC	Right PFC	Left PFC	Right PFC	Target		Left PFC	Left PFC
MI	0.4	0.8	0.4	0.8	MI		0.4	0.8
Definity microbubble dose	1x	1x	1x	1x	Definity microbubble dose		1x	1x
MRI 1—BBB opening—safety	Day 0		Day 0		MRI—BBB opening—safety		Day 0	Day 0
MRI 2—BBB closing—safety	Day 1		Day 3		Behavioral		Days 1–23	Days 1–21
Euthanasia	Day 2		Day 18		Euthanasia		N/A	N/A
Age	8 y		7 y		Age		7 y	8 y

### FUS treatment

In preparation for the FUS procedure using the clinical setup, the animal was sedated, intubated, and an intravenous catheter was placed in the saphenous vein. The NHP was then anesthetized with isoflurane (1–2%) and oxygen delivered through the endotracheal tube. The hair on the targeted region of the scalp was removed by shaving and application of depilatory cream. The shaved section was covered with a non-toxic, water-soluble coupling gel to ensure efficient transmission of the ultrasound waves. The FUS procedure lasted for approximately 30 min. It involved targeting using the neuronavigation system, delivery of Definity microbubbles injected through a catheter, and then applying FUS to the pre-planned target for BBB opening. The scalp and skull were left intact during this process. We recorded the microbubble acoustic emissions and calculated the cavitation doses in real-time (see Supplementary methods).

**Figure 1:**
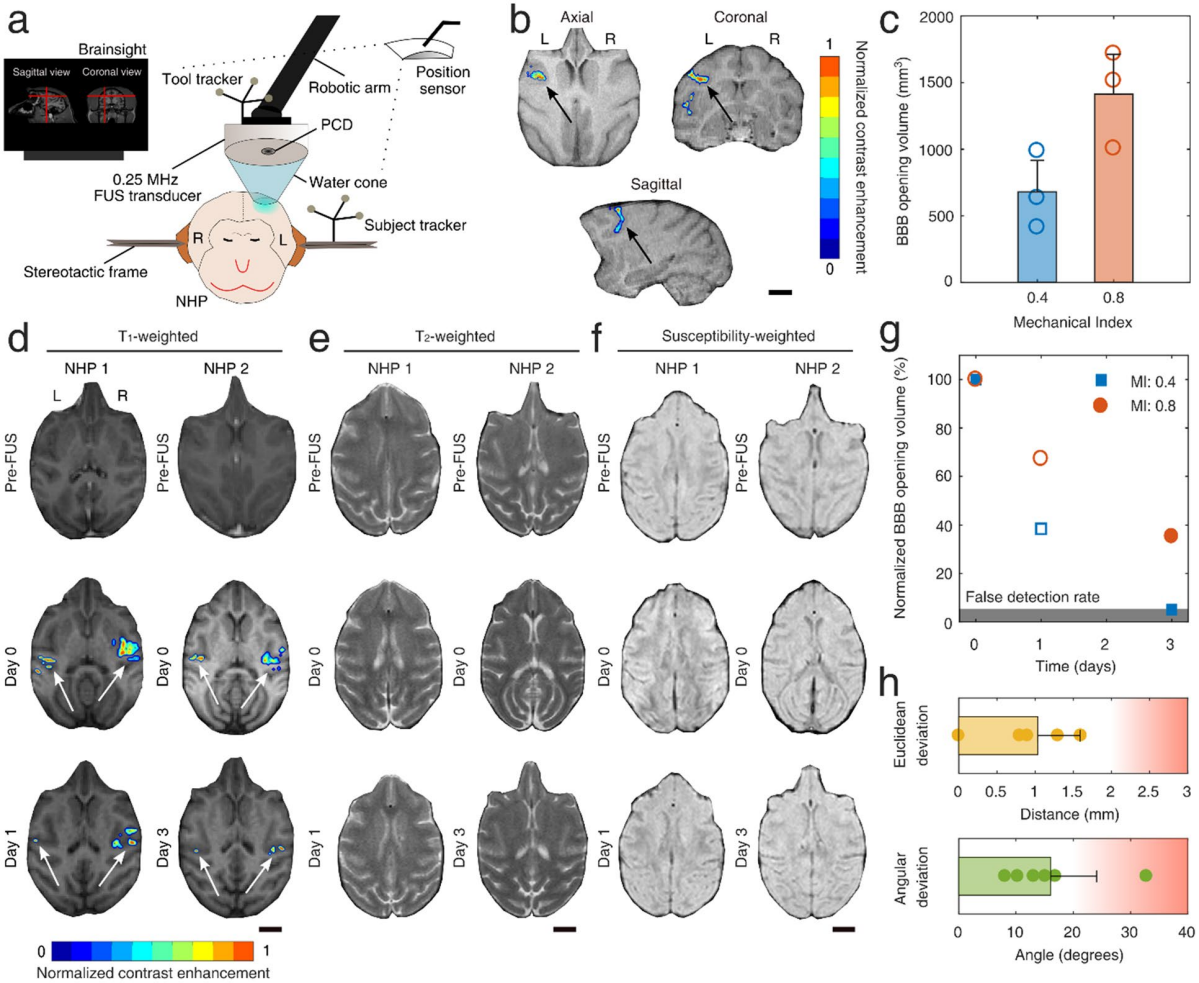
Targeted blood–brain barrier opening in non-human primates using focused ultrasound and microbubbles. (**a**) Neuronavigation-guided FUS system. A 0.25-MHz transducer was attached to a robotic arm and positioned above the NHP head. The T_1_-weighted MRI acquired during treatment planning was loaded onto the Brainsight neuronavigation system and was used to guide the FUS treatment. An infrared position sensor located the subject and tool trackers in real-time, guiding the placement of the FUS focal volume within the pre-planned area. (**b**) Contrast-enhanced T_1_-weighted MRI showing areas with BBB opening (colored ROI) in the prefrontal cortex of NHP 3 (MI: 0.4), along the axial, coronal, and sagittal planes. Color bar: normalized contrast enhancement. (**c**) BBB opening volume for MI of 0.4 and 0.8 (n = 3 per condition). (**d**) T_1_-weighted MRI before FUS (top row), 1 h post-FUS (middle row), and day 1 or 3 (bottom row) for NHPs 1 and 2, respectively. Left (L) side was treated with MI of 0.4, while the right (R) side was treated with MI of 0.8. Color bar: normalized contrast enhancement. (**e**) T_2_-weighted MRI before FUS (top row), 1 h post-FUS (middle row), and day 1 or 3 (bottom row) for NHPs 1 and 2, respectively. (**f**) Susceptibility-weighted MRI before FUS (top row), 2 h post-FUS (middle row), and day 1 or 3 (bottom row) for NHPs 1 and 2, respectively. (**g**) BBB closing timeline expressed as the percentage of disrupted volume at day 0 remaining permeable at days 1 (NHP 1, empty symbols) and 3 (NHP 2, filled symbols). Gray area corresponds to the false detection rate (5%), indicating pixels whose value randomly fluctuates above the detection threshold. (**h**) Robotic arm accuracy. Euclidean (top panel) and angular (bottom panel) deviation between planned and achieved focal volume placement. The values were acquired from BrainSight software 2.4 (www.rogue-research.com). Scale bars indicate 1 cm. FUS, focused ultrasound; NHP, non-human primate; BBB, blood–brain barrier; PCD, passive cavitation detector; MI, mechanical index.

**Figure 2:**
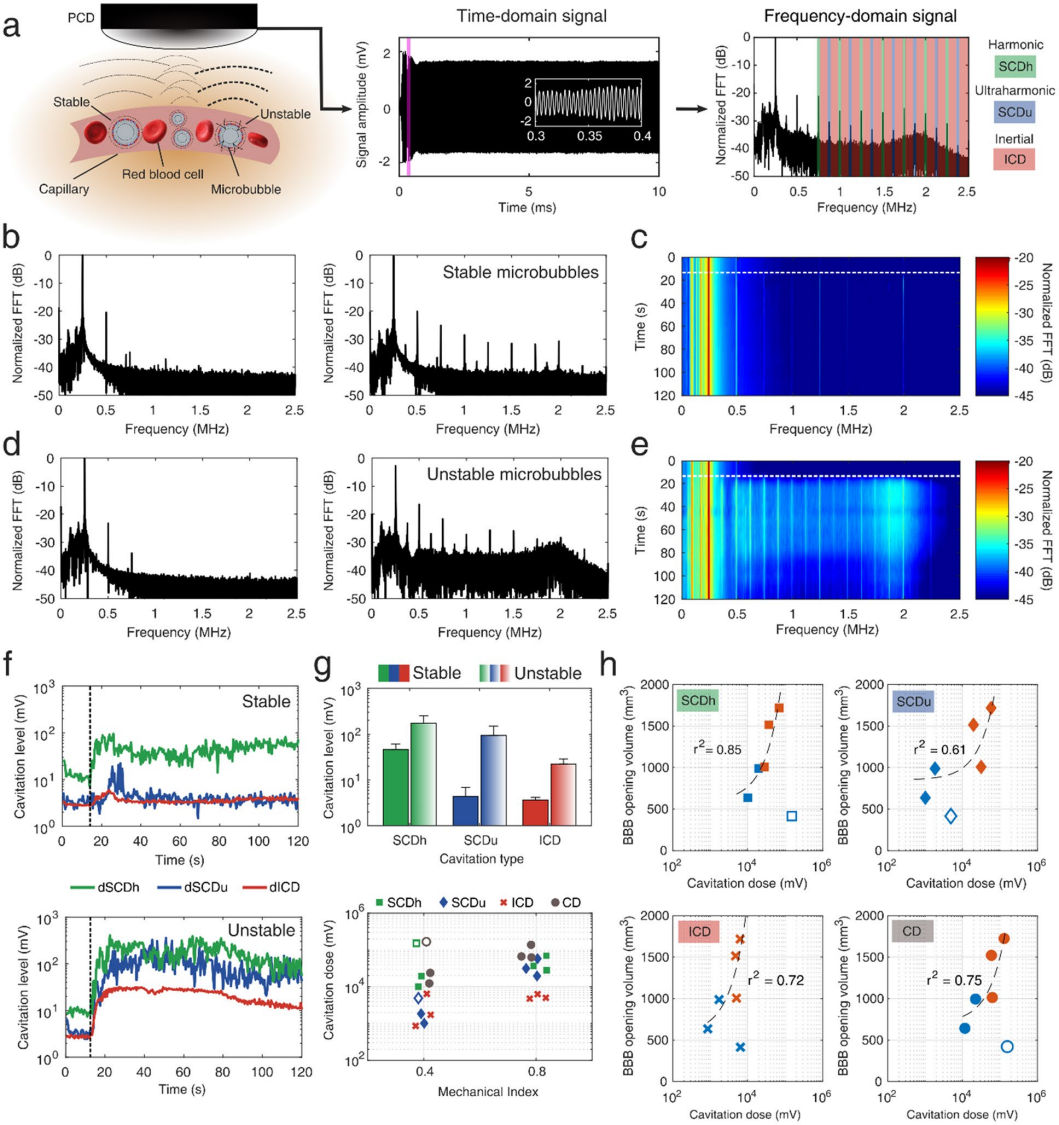
Real-time monitoring of blood–brain barrier opening through passive cavitation detection. (**a**) Microbubbles exposed to FUS re-radiate their own acoustic emissions which can be captured through a single-element PCD. Microbubbles can oscillate either in a “stable” and recurrent manner (low pressure or MI of 0.4), or in an “unstable” and violent manner (high pressure or MI of 0.8), which may lead to their fragmentation and jet formation. The latter oscillation mode radiates higher amount of acoustic energy. The time-domain signal had duration of 10 ms, recording the microbubble response throughout the therapeutic pulse. White inset corresponds to the purple area in the beginning of the pulse. Time domain signal was converted to the frequency domain by calculating the FFT. Spectral domains containing the harmonics (green areas), ultraharmonics (blue areas), and broadband (red areas) emissions were isolated to calculate the respective cavitation levels and doses. (**b**) Example spectra before (left) and after (right) the intravenous injection of microbubbles, during FUS treatment at MI of 0.4. (**c**) Spectrogram including the frequency response throughout the 2-min treatment duration at MI of 0.4. (**d**) Example spectra before (left) and after (right) the intravenous injection of microbubbles, during FUS treatment at MI of 0.8. (**e**) Spectrogram including the frequency response throughout the 2-min treatment duration at MI of 0.8. (**f**) Cavitation levels throughout the FUS treatment at MI of 0.4 (top panel) and 0.8 (bottom panel). Harmonic stable cavitation levels (green line) dominated during treatment at MI of 0.4. Ultraharmonic stable (blue line) and inertial (red line) cavitation levels rose only during treatment at MI of 0.8. (**g**) Top: average cavitation levels for FUS treatment at MI of 0.4 (filled bars) and 0.8 (patterned bars). Bottom: Harmonic stable, ultraharmonic stable, inertial, and total cavitation dose for FUS treatment at MI of 0.4 and 0.8. (**h**) Correlation between BBB opening volume and harmonic stable (squares), ultraharmonic stable (diamonds), inertial (crosses), and total (circles) cavitation dose. Light blue and orange symbols denote treatment and MI of 0.4 and 0.8, respectively. Harmonic stable cavitation dose had the highest correlation with the resulting BBB opening volume (r^2^ = 0.85). Cavitation levels were calculated for each individual therapeutic pulse, while cavitation dose was the sum of all cavitation levels throughout the 2-min FUS treatment. Empty symbols in (**g**) and (**h**) correspond to an outlier with low signal-to-noise ratio (SNR ~ 1; NHP 3). Dotted lines in (**c**), (**e**) and (**f**) denote the time point in which microbubbles enter the focal volume, following their intravenous administration at t = 0 s. Data in (**g**) are presented as mean ± standard deviation (n = 210 pulses after microbubble signal detection). PCD, passive cavitation detector; MI, mechanical index; FFT, fast Fourier transform; dB, decibel; dSCDh, stable cavitation level based on harmonic emissions; dSCDu, stable cavitation level based on ultraharmonic emissions; dICD, inertial cavitation level; SCDh, stable cavitation dose based on harmonic emissions; SCDu, stable cavitation dose based on ultraharmonic emissions; ICD, inertial cavitation dose; CD, total cavitation dose.

The ultrasound transducer was remotely moved by a 4-degrees-of-freedom robotic arm (Kinova Jaco^2^, Kinova, Boisbriand, QC, Canada). FUS propagated within a cone filled with degassed and distilled water. Definity microbubbles were injected intravenously at the clinically-recommended dose (10 μl/kg). Microbubbles under FUS excitation oscillate within the cerebral microvessels leading to a reversible BBB opening through mild mechanical stimulation^8^. Detailed description of the focused ultrasound setup (Fig. 1a and Fig. S1), the acoustic cavitation analysis (Fig. 2 and Fig. S6), and the BBB opening quantification graphical user interface (Fig. S14) can be found in the Supplementary material.

#### MRI

Pre-FUS MRI was used for planning purposes. A set of fiducial markers were attached to the NHP’s tooth line using a thermoplastic material. These markers were registered in the BrainSight software 2.4 (Rogue Research, Montreal, QC, Canada) to allow for accurate placement of the ultrasonic focus to the targeted structure through neuronavigation-guidance^62,64^. We used MRI with and without contrast to observe BBB opening and monitor for potential trauma induced by FUS. NHPs remained sedated between the ultrasound procedure and the MRI. Immediately after the ultrasound procedure, we performed an MRI scan with a contrast agent to assess the permeability of the BBB in the region that was targeted by the FUS. During scans, fluids were supplied intravenously, and vital signs such as pulse, respiration rate, and SpO_2_ were monitored by MR-compatible monitoring equipment. Electrocardiography (ECG) was used in conjunction with routine MRI procedures and monitoring. ECG was conducted via a system built into the MRI room. Animals were monitored continuously and were physically checked in between sequences. The animal was covered with a blanket and insulating material to preserve body temperature during each scan.

For all scanning procedures, the NHPs were anesthetized with an initial dose of ketamine and, if needed, propofol. Isoflurane or IV propofol anesthesia was used throughout the scanning. The NHP was positioned on the MRI scanning table before the scan. The MRI scan was acquired using a standard RF head coil. The MRI sequences acquired were T_1_-weighted, T_2_-weighted and susceptibility-weighted (SWI), to assess the BBB opening, edema, and hemorrhage, respectively. A scale bar for size calibration and fiducial markers for the head position visualization was used in the pre-FUS MRI to improve co-registration with skull landmarks. The total time on the MRI scanning table was less than 2 h. More details on the MRI sequences used for this study can be found in the Supplementary methods.

### Neurobehavioral examinations

Observational neurological examinations were performed daily by a veterinarian experienced with NHPs, based on a quantitative scoring system (Table S7). Examinations were performed either in the animal’s home cage or adjacent to their home cage in an attached and familiar play cage. Initially, the animal was observed for 1–2 min to assess mentation and posture. Enrichment food items were then offered in multiple positions to encourage head and eye movement, ambulation of all four limbs, and fine motor movements. This allowed assessment and semi-quantitative grading of visual tracking (Cranial nerves [CN] III, IV, and VI), pupil size and reflex (CN II and III), ambulation and proprioception, and fine motor movements. Facial muscle movement and tone (CN VII) were also assessed during this time, while enrichment was fed. These values were evaluated on a scale of 0 (absent), 1 (depressed), 2 (normal), or 3 (hyper-reactive), on both the right and left sides. Appetite and interest in enrichment were also assessed. Any abnormalities during the examination or daily health checks were noted.

### Histological analysis

NHPs 1 and 2 were euthanized through transcardial perfusion (see Supplementary methods for euthanasia protocol), and their brains were extracted and processed for histology. Prior to histology preparation, each fixed brain was fixed in 4% paraformaldehyde (PFA). The brains were coronally sectioned into 2 mm slices, from an anterior to posterior direction. Sections were halved into right and left hemispheres and blocked in cassettes to be embedded in paraffin. They were then prepared for slides and further sectioned at 5 μm for staining.

We evaluated the immunogenic and neurogenic effects of FUS therapy through immunohistochemistry. Microscopic damage and morphological changes were assessed with hematoxylin and eosin (H&E), enhanced with luxol fast blue, to aid in anatomical visualization by highlighting fiber tracts. H&E was used to assess necrosis, hemorrhage, and blood vessel integrity. We also stained for astrocytes using glial fibrillary acidic protein (GFAP). To further evaluate short-term and long-term immune response, we stained for microglia using ionized calcium-binding adapter molecule 1 (Iba1) and cluster of differentiation 68 (CD68), in order to observe the presence of microglia as well as macrophages. Both Iba1 and CD68 are considered markers for microglia and macrophages; Iba1 is expressed in both resting and activated microglia, and CD68 is a lysosomal protein that is highly expressed in macrophages and microglia and less expressed in resting microglia. Iba1 and CD68 do not necessarily overlap^65^, so the union of the two stains was used to quantify the presence of microglia and macrophages in perivascular areas (Fig. S7). Bielschowsky’s silver stain was performed to visualize neuronal cell processes, axons, and neurofilaments. Signs of neurogenesis within the treated areas were assessed with doublecortin (DCX)^66^. The number of DCX^+^ cells was recorded at regions of interest within areas that underwent BBB opening and outside the treated areas (n = 5 per hemisphere and time point). The BBB opening regions for DCX imaging were chosen based on the contrast-enhanced T_1_-weighted imaging, i.e. we imaged only non-overlapping areas with contrast enhancement. In contrast, areas outside the focal area which did not have contrast enhancement were randomly chosen to derive the baseline cell density. We did not perform registration between the brain slices and MRI scans. However, the corresponding MRI slices were chosen based on anatomical similarity with the histology slices.

Brain sections were also stained for caveolin 1 and TUNEL to evaluate the extent of caveolin-mediated endocytosis and FUS-triggered apoptosis, respectively. Slices were examined by a trained neuropathologist, blinded to the experimental conditions, and by researchers with experience in histology. We obtained microscopy images with variable magnification. The primary target of the analysis on the tissue level was to evaluate gross qualitative histological changes in the exposed tissue compared to non-exposed regions. The exposed cortical and subcortical regions were chosen based on the contrast-enhanced T1-weighted MRI. We also qualitatively evaluated cellular morphology wherever possible. However, quantification of cellular morphology was beyond the scope of the current study. More details on the histological analysis, i.e. immunohistochemistry with Iba1-CD68, GFAP, DCX, and Cav1, and cell counting methods (Figs. S7 and S8), can be found in the Supplementary material.

### Behavioral testing

Lesions within the targeted PFC areas have been previously correlated with cognitive decline and behavioral change in NHPs^67–69^. Damage in frontal networks produces deficits in behavioral initiation, attention, motivation and motor function^69^. We hypothesized that these executive functions would be affected in the case of FUS-induced damage, leading to cognitive performance compromise. NHPs 3 and 4 had been trained to a behavioral test assessing transitive inference performance^60^. Transitive inference is related to the ability to infer that A > C, given that A > B and B > C. This test assessed for inference capacity, spatial memory, reaction time, cognition, and decision making. Both NHPs had reached the peak of their learning curve prior to this study. The task involved presenting 2 images drawn from a new list of 7 images, which was different every day (Fig. 4b). Presentation of entirely novel images every day ensured that NHPs were not over-trained on a particular stimulus set, but rather acquired mastery of the general task. Each image had a variable implicit value. A reward was given to NHPs through the fluid dispenser when they selected the image with the higher implicit value. No reward was given for erroneous or no answers. NHPs performed the test daily in their home cage (Fig. 4a) for at least 28 days before the FUS treatment and for at least 21 days after FUS treatment. There was no behavioral testing on the day of FUS treatment (i.e., day 0) to avoid confounding effects induced by anesthesia. NHPs performed the task the day before and the day after FUS (i.e., days − 1 and 1). Performance was assessed in terms of accuracy (fraction of correct answers to total number of answers or choice trials) and mean reaction time (natural logarithm of reaction time in ms). Responses given by pure chance would result to an accuracy of 0.5. Accuracy and reaction time were also analyzed in terms of the target location, i.e. left-ipsilateral vs. right-contralateral, and the test phase, i.e. training phase vs testing phase. Training phase included trials 1–120, while testing phase included trials 121 to 540. We included both training and testing phase data in the average accuracy and reaction time calculation, to avoid bias due to the arbitrary threshold selection between training and testing phases. Due to limited availability of the required FUS-naïve NHPs, a sham FUS condition was not included in the experimental design. Based on our previous studies, sham FUS sessions had no significant effect in cognitive performance^54,55^.

### Statistical analysis

Averaged measurements reported in this study are expressed as mean ± standard deviation, unless otherwise stated. Results are shown for a total of n = 6 FUS treatments, n = 3 at MI of 0.4 and n = 3 at MI of 0.8 in 4 different NHPs (Table 1). To reduce compounding factors and identify effects from a single FUS treatment, none of the NHPs was exposed to FUS before the reported experiments and treatments were performed in non-overlapping areas of either hemisphere in NHPs 1 and 2. BBB opening volume was correlated with cavitation dose through linear regression and the corresponding coefficient of determination r^2^. Response accuracy and reaction times were modeled using logistic regression, predicting performance in terms of day, target position, and testing phase. The parameters from the logistic regression analysis were calculated in both the pre- and post-FUS periods, to investigate the performance trend during each period. The logistic regression analysis of NHP 3 was conducted independently from NHP 4. Performance on the day of FUS was inferred by calculating the intercept of the logistic regression in both periods on day 0. We calculated the mean estimates and 95% confidence interval for the mean on day 0 pre- and post-FUS. Comparing intercepts provided an assessment of the step change occurring due to the FUS exposure on day 0.

## Results

### FUS induces safe BBB opening in NHPs

Non-invasive and targeted BBB opening in four NHPs was performed using a single-element neuronavigation-guided FUS system (Fig. 1a and Fig. S1). Anesthetized NHPs were treated with FUS, targeting an area within the PFC (Fig. S1a). BBB opening was observed within the PFC, primarily between the arcuate and central sulci (Fig. 1b), but also within other areas such as the pre-motor and primary motor cortex. Due to the large focal volume of the clinical transducer (i.e., 6 mm × 6 mm × 49 mm)^62^ and limited angle control imposed by the 4-degree-of-freedom robotic arm, the BBB opening expanded well beyond the focal point, covering multiple cortical and sub-cortical areas. BBB was disrupted in all treatments at both pressures tested. BBB opening volume depended on the MI (Fig. 1c), and was measured to be 680 ± 236 mm^3^ at MI of 0.4 (n = 3), and 1413 ± 299 mm^3^ at MI of 0.8 (n = 3). This calculation was performed on all available axial MRI slices for each NHP (n = 71 slices). NHPs 1 and 2 were bilaterally treated (Fig. S2a) and were allowed to survive for 2 and 18 days, respectively, to assess short- and long-term FUS-induced effects (Table 1). We first opened the BBB in the left hemisphere (MI: 0.4), followed by the right hemisphere (MI: 0.8) 30 min later. This interval was selected to allow complete clearance of the first microbubble bolus from circulation (microbubble half-life: 1.3–1.9 min). MRI scans were acquired within a maximum time interval of 1–2 h after the final FUS procedure.

Contrast-enhanced T_1_-weighted MRI confirmed BBB opening in both treated brain regions for NHPs 1 and 2 (Fig. 1d). FUS at low pressures produced a BBB opening within the left PFC, mainly within the gray matter. FUS at high pressures resulted in more substantial contrast enhancement in both gray and white matter within the right PFC compared to the left PFC. BBB opening was observed throughout the gray matter in both NHPs at the highest MI, confirming previous studies investigating the BBB opening distribution within the white and gray matter of NHPs^70^. Ultrasound reflections at the skull/tissue interface potentially increased the local pressure due to interference and gave rise to limited BBB opening at the outermost part of the frontal and parietal lobes (Fig. S3). Surface effects were expected but were preferred over the potential overlap of the treated regions.

To evaluate the BBB reinstatement rate, we performed contrast-enhanced T_1_-weighted MRI at days 1 (NHP 1) and 3 (NHP 2). The BBB closing timeline was constructed by combining data from both NHPs 1 and 2 (Fig. 1g). BBB opening within the white matter was restored on day 1 for NHP 1. We observed a punctate distribution of BBB-opened regions within the gray matter of the left PFC (MI: 0.4) that had not been completely restored on day 1 (Fig. 1d-bottom row). In sharp contrast, there remained extensive BBB opening in the right PFC (MI: 0.8), especially at the center of the focus and across the brain/skull interface. By day 3, we did not observe any region with BBB opening in the left PFC, except for a few sporadic pixels that surpassed the noise threshold. At the same time, the right PFC remained open primarily in its superior part close to the FUS beam’s entry point. The BBB opening percentages of the initial volumes were equal to: 38.4% on day 1 and 5% on day 3 for MI of 0.4; and 67.3% on day 1 and 35.3% on day 3 for MI of 0.8.

T_2_- (Fig. 1e and Fig. S4) and susceptibility-weighted (SWI; Fig. 1f and Fig. S5) MRI scans were performed to establish the radiological safety profile of the FUS treatment on days 0, 1, and 3. All scans were qualitatively compared to their baseline, acquired before the FUS treatment (Fig. 1e, f, top row). There was no hemorrhage or any acute damage observable with T_2_ or SWI sequences on days 0 and 1, indicated by the absence of asymmetric or new hyper-intense or hypo-intense regions, respectively. We did observe a minute hyper-intense area in the T_2_ scan of NHP 2 on day 3, corresponding to the right PFC (MI: 0.8), which was not present in the baseline scan. The hyper-intense area (2 × 2 mm^2^, white arrow in Fig. S4) was constrained within two axial slices of the superior motor cortex. However, there was no hypo-intense area in the respective SWI slice (Fig. S5). Also, there was a region within the lateral cortex of NHP 1 on day 1, with moderately higher T_2_ signal in the right hemisphere compared to day 0 (Fig. S4), which may indicate a potential edema.

In general, the robotic arm performance was within the acceptable limits^64,71^, in terms of the distance from the intended focus and the angular deviation from the planned trajectory (Fig. 1h and Fig. S2d). The curvilinear brain (Fig. S2b) and skin reconstructions (Fig. S2c), in combination with the bull’s eye view function of the BrainSight platform (Fig. S1b), allowed fast targeting (< 10 min). The average targeting error in Euclidean distance was 1.03 ± 0.55 mm (n = 6). The average angular deviation from the planned trajectory was 14.92° ± 9.43°, caused by the 4 degree-of-freedom robotic arm’s limited angle range. These values were obtained from the BrainSight platform in real-time and were not derived by the BBB opening distribution, which was diffuse, especially at low pressures. The Euclidean error and angular deviation shown here represent the restraints induced by the limited control of the robotic arm, which lead to BBB opening away from the intended target.

### FUS treatment can be acoustically monitored in real-time

One of the distinct advantages of most FUS therapies is the ability to monitor their evolution in real-time through transcranial passive cavitation detection (PCD). Our clinical system was equipped with a 1.5-MHz PCD transducer co-aligned with the 0.25-MHz FUS transducer (Fig. 1a and Fig. S1a). Microbubble emissions from within the vasculature (Fig. 2a-left) were captured with the PCD transducer, recorded through an acquisition card, and plotted in the time- (Fig. 2a-center) and frequency- (Fig. 2a-right) domain in real-time. Cavitation emissions were also monitored in terms of stable harmonic, stable ultraharmonic, inertial, and total cavitation dose (SCD_h_, SCD_u_, ICD, and CD, respectively), following isolation of the respective spectral regions (Fig. 2a-right). More details on the acoustic analysis can be found in the Supplementary methods.

Treatment at low pressures triggered stable and recurrent microbubble oscillations, indicated by the emergence of pure harmonic emissions, which were not present in the control sonication (Fig. 2b). Harmonic emissions persisted throughout the 2-min treatment duration (Fig. 2c). In contrast, treatment at high pressure resulted in unstable microbubble oscillations or “inertial” cavitation, indicated by the increased broadband acoustic emissions, alongside strong ultraharmonic emissions (Fig. 2d). Inertial cavitation of high amplitude persisted for approximately 60 s, followed by inertial cavitation of reduced amplitude (Fig. 2e). Cavitation response over time was quantified by calculating cavitation levels (i.e., dSCD_h_, dSCD_u_, and dICD—Supplementary methods), which rose smoothly with the increase of microbubble concentration within the focal volume (Fig. 2f-top). At low pressures, dICD remained equal to baseline, without any sustained rise throughout the 2-min FUS treatment. Similarly, dSCD_u_ remained constant except for several short-lived bursts. Increasing the acoustic pressure produced a different cavitation response, characterized by an increase of the dICD by an order of magnitude, which remained present until the end of the FUS treatment (Fig. 2f-bottom). dSCD_h_ and dSCD_u_ increased by a factor of 20 compared to baseline. Cavitation doses followed a decreasing trend over time (Fig. S6). This may be correlated with the clearance time of Definity microbubbles, which are halved in concentration within 2 min after their IV administration. Qualitatively, treatments at both low and high acoustic pressures with an acceptable signal-to-noise ratio had similar frequency responses (Fig. S6a, S6b) and cavitation dose evolution (Fig. S6c, S6d).

Stable harmonic cavitation levels during treatment (dSCD_h_) were on average 4 times higher at high pressure than low pressure (Fig. 2g-top). The change in stable ultraharmonic (dSCD_u_) and inertial cavitation (dICD) levels was higher, presenting a 50-fold and 7-fold increase, respectively. The total cavitation dose (CD) was on average 10 times higher at MI of 0.8 than at MI of 0.4 (Fig. 2g-bottom). BBB opening volume had a strong correlation with the stable harmonic (SCD_h_) cavitation dose (r^2^ = 0.85—linear regression; Fig. 2h). Stable ultraharmonic, inertial, and total cavitation dose were also correlated with the BBB opening volume, albeit presenting lower coefficients of determination (r^2^ = 0.61 for SCD_u_, r^2^ = 0.72 for ICD, and r^2^ = 0.75 for CD).

### Histological observations following FUS-mediated BBB opening

**Figure 3:**
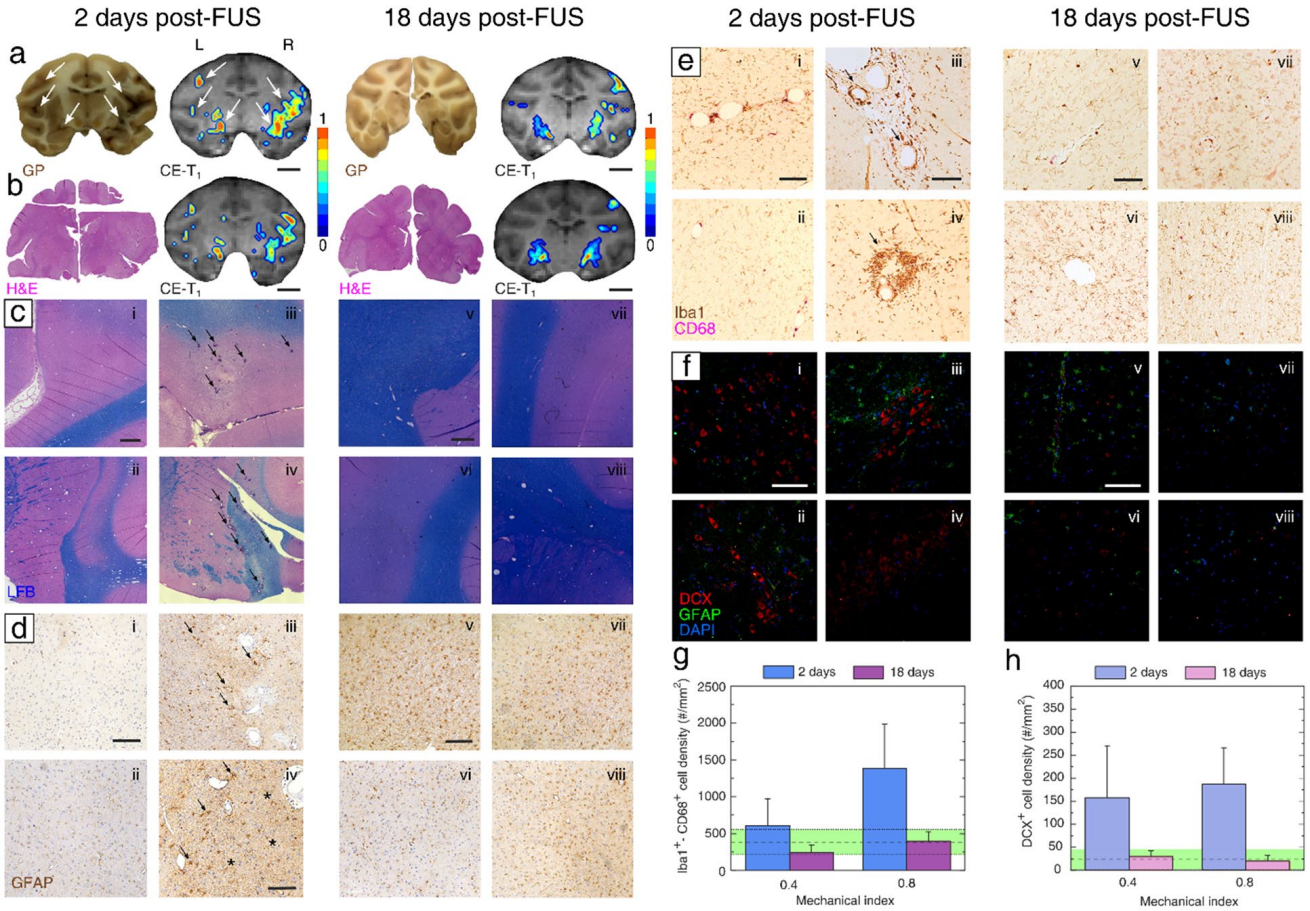
Histopathological analysis of the non-human primate brain tissue following focused ultrasound treatment. (**a**) Gross pathology and corresponding BBB opening area at the 2-day (left) and 18-day (right) time point. (**b**) H&E staining and corresponding BBB opening area at the 2-day (left) and 18-day (right) time point. (**c**) Luxol fast blue H&E staining for myelin delineation at the 2-day (i–iv) and 18-day (v–viii) time point. (**d**) GFAP staining for astrocyte presence at the 2-day (i–iv) and 18-day (v–viii) time point. (**e**) Iba1/CD68 staining for microglia presence and migration at the 2-day (i–iv) and 18-day (v–viii) time point. (**f**) DCX staining for immature neurons at the 2-day (i–iv) and 18-day (v–viii) time point. GFAP and DAPI were used to stain astrocytes and cell nuclei, respectively. (**g**) Iba1^+^ and CD68^+^ cell density within perivascular areas treated at MI of 0.4 and 0.8, examined at the 2-day (deep blue bars) and 18-day (purple bars) time points. (**h**) DCX^+^ cell density with brain regions treated at MI of 0.4 and 0.8, examined at the 2-day (light blue bars) and 18-day (pink bars) time points. Regions of interest at (**c**), (**d**), (**e**), and (**f**) were within the left hemisphere treated at MI of 0.4 (i, ii, v, vi) or within the right hemisphere treated at MI of 0.8 (iii, iv, vii, viii). Color bars: normalized contrast enhancement. Scale bars: 1 cm (**a**, **b**); 1 mm (**c**); 200 μm (**d**, **e**); 100 μm (**d**-iv, **e**-iii, f). All images shown here correspond to regions of interest within areas with BBB opening as confirmed by MRI. Green shaded areas in (**g**) and (**f**) correspond to baseline Iba1^+^-CD68^+^ and DCX^+^ cell density, in brain areas outside the treated volume. Data in (**g**) and (**h**) are presented as mean ± standard deviation (n = 5 regions of interest per section and per hemisphere, n = 2 sections). GP, gross pathology; H&E, hematoxylin and eosin; CE-T_1_, contrast-enhanced T_1_ MRI scan; LFB, luxol fast blue for myelin delineation; GFAP, glial fibrillary acidic protein; Iba1, ionized calcium binding adaptor molecule 1; CD68, cluster of differentiation 68; DCX, doublecortin; DAPI, 4′,6-diamidino-2-phenylindole.

NHPs 1 and 2 were euthanized through transcardial perfusion on day 2 and 18 post-FUS, respectively. Their brains were extracted and processed for histology. Following microscopic examination, we identified a mechanical effect in the right hemisphere (high pressure—MI: 0.8) of NHP 1 two days post-FUS, observed as a dark region in the lateral cortex, possibly indicative of fluid accumulation and edema formation (Fig. 3a). The dark regions overlapped with the regions of high-volume BBB opening. H&E staining enhanced with luxol fast blue (Fig. 3b, c) indicated red blood cell extravasation in perivascular areas within the BBB opening volume (arrows—Fig. 3c-iii, c-iv). The left hemisphere (low pressure—MI: 0.4) appeared intact and free of effects on perivascular areas (Fig. 3c-i, c-ii). We observed a number of GFAP^+^ cells (i.e., astrocytes) within the exposed area (Fig. 3d-iii, iv). There were instances of astrocytes with higher GFAP intensity (arrows in Fig. 3d-iv) compared to their neighboring cells (stars in Fig. 3d-iv). Microglia and macrophages shown by Iba1-CD68 staining were concentrated in the periphery of blood vessels of the right hemisphere (Fig. 3e-iii, e-iv), suggesting enhanced migration and/or proliferation towards the impacted vessels^72^. Microglia were observed to have thickened processes as well as enlarged bodies. Doublecortin-positive (DCX^+^) cells were detected within the treated areas of both the left (Fig. 3f.-i, f-ii) and the right (Fig. 3f.-iii, f-iv) hemisphere, indicating signs of increased number of immature neurons triggered by FUS.

In contrast, there was no sign of long-term increase in microglia density or any observable histological change at a tissue level in NHP 2, euthanized 18 days post-FUS. Both hemispheres appeared intact visually on gross examination (Fig. 3a), while H&E staining revealed no microscopic hemorrhage, necrosis or abnormal cell morphology (Fig. 3b, c-v through c-viii). GFAP staining showed an absence of gliosis; GFAP-positive cells qualitatively appeared with no abnormal morphology within the targeted area (Fig. 3d-v through 3d-viii). Iba1 and CD68 staining showed no abnormal accumulation of macrophages or microglia in the vessel periphery in either hemisphere (Fig. 3e-v through e-viii). Finally, there was a limited number of low-signal DCX^+^ cells in both hemispheres (Fig. 3f-v through 3f-viii), whose density was not higher from baseline density in non-treated areas. All images shown in Fig. 3 were taken from regions which underwent BBB opening as confirmed by contrast-enhanced T1-weighted MRI.

We estimated the average density of microglia within the BBB opening area (Fig. S7 and Supplementary methods). Due to the spectral overlap of the Iba1 and CD68 chromogens, bright-field images were converted into grayscale and thresholded to identify monocyte lineage cells. Both Iba1 and CD68 are typically used to identify microglia; however, they do not always overlap, with Iba1 being a more suitable marker for morphology estimation in the absence of pathology and CD68 reflecting an immune activation and response to tissue damage^65^. Therefore, both Iba1^+^ and CD68^+^ cells were taken into account to identify microglia and/or macrophages. Two days after treatment, there was an increase of Iba1^+^-CD68^+^ cells in the right hemisphere (MI: 0.8) compared to the baseline areas outside the focal volume (Fig. 3g). This effect was driven by the accumulation of such cells in the vicinity of blood vessels, which were likely undergoing repair (Fig. 3e-iii, e-iv). There was a moderate increase of cell density in the left hemisphere (low pressure—MI: 0.4), however not different than baseline. Notably, both treatment conditions did not considerably change microglia density 18 days post-FUS, compared to baseline.

The observed increase in the number of immature neurons was quantified by estimating the DCX^+^ cell density (Fig. S8) within the treated areas and in control regions that did not undergo BBB opening (Fig. 3h). DCX imaging was focused on areas which presented contrast-enhancement in T_1_-weighted imaging following BBB opening (Fig. 3a, b, white arrows). A relative increase in the density of immature neurons was observed in both the left and the right hemispheres, compared to baseline. DCX^+^ cells were observed within the dorsolateral PFC (6DC, 6VC, and 8A areas, within the principal, arcuate, and non-principal sulci), the thalamus, sub-thalamic nucleus, and the hippocampus. These effects were observed 2 days post-FUS. In contrast, there was no considerable change of the DCX^+^ cell density in either hemisphere 18 days after treatment. Although different brain areas are expected to have a variable number of innate DCX^+^ cells, averaging was performed in multiple neurogenic and non-neurogenic areas with and without BBB opening to acquire an average density approximation. Merging of multiple areas is a confounding factor that should be considered when interpreting the quantitative outcomes of the histological analysis. The measured cell density does not represent the density within the entire focal area, but instead the localized density of the observed groups of DCX^+^ cells (Fig. 3f).

We detected signs of axonal and cell body injury in the silver stain within the impacted area of the right hemisphere at the 2-day time point (Fig. S9). Similarly, TUNEL^+^ cells indicated apoptosis triggered by FUS exposure at high MI (Fig. S10). These effects were constrained within the perivascular areas exposed to ultrasound at MI of 0.8, and were not present in the left hemisphere at the 2-day time point or in either hemisphere at the 18-day time point. Caveolin-1 appeared to be upregulated within the endothelial cells surrounding arterioles and capillaries at the 2-day time point after treatment at MI of 0.4 (Fig. S11). This indicates that caveolin-mediated endocytosis may be a potential mechanism of uptake following low-pressure FUS treatment.

These results demonstrate that at the tissue level, high-pressure FUS treatment triggered increased concentration of glial cells on day 2 (n = 1 NHP), which was no longer detected on day 18 (n = 1 NHP). Furthermore, FUS-mediated BBB opening led to an increased occurrence of DCX^+^ cells on day 2 (n = 1 NHP) post-FUS compared to day 18 (n = 1 NHP). Our analysis assumed that the initial histological effects would be similar between NHP 1 and NHP 2. This hypothesis was supported by the similar BBB opening volumes (Fig. 1) and the similar cavitation response at both acoustic pressures (Fig. 2) for both animals.

### Blood chemistry and neurobehavioral traits are not affected by BBB opening

To assess potential biochemical effects of the FUS-induced BBB opening, we performed blood tests before FUS (baseline), 2 days post-FUS (short-term effects), and 18 days post-FUS (long-term effects). Complete blood count showed an increase in reticulocytes (from 0.45% to 27.34%) and platelets (from 390 × 10^3^/μl to 702 × 10^3^/μl) 2 days post-FUS for NHP 1 (Table S1). All other cells had little or no change compared to the baseline. Reticulocytes are immature red blood cells under development and platelets are an essential clotting requirement. The increase of these two populations may be correlated with the BBB restoration process (Fig. 1d, g). Both reticulocytes and platelets were within the normal range for NHP 2, when examined 18 days post-FUS (Table S2). All blood metrics presented typical values, except for mean corpuscular hemoglobin concentration, which was marginally lower than the minimum range. However, the hematopoietic system is not likely to be affected by the FUS procedure. Thus, we can conclude that there is no long-term effect on the blood count values post-FUS.

In terms of cell count, NHP 1 presented an increase of eosinophils (from 0.6% to 35.26%) on day 2 (Table S3). Eosinophils are a type of white blood cell that is important in normal physiological function and host defense. They are often involved in allergic and inflammatory responses, and are particularly important in immune responses to parasitic infection. Their temporary increase may be related to the BBB opening, which facilitates blood protein diffusion into the parenchyma (e.g., albumin), eliciting a short-lived immune response^39,74^. Alternatively, eosinophilia may be a reaction to drugs used during anesthesia induction and maintenance. All cell count values were within the normal range for NHP 2 on day 18 (Table S4). Blood chemistry values were within the normal range at both 2 and 18 days post-FUS (Tables S5 and S6). The only elevated value was the 2-day level of alanine transaminase (ALT), a liver enzyme involved in cellular energy production (Table S5). Elevated levels often indicate hepatocellular damage, but there were no other indications of liver disease. ALT is not hepatocyte-specific and can be released from other cell types, including skeletal and cardiac muscle. This elevation is unlikely to be due to the opening of the BBB. Instead, it is more likely that ketamine used for anesthesia induction or circulating Definity microbubbles caused the short-term ALT elevation. In conclusion, there was no considerable long-term change in complete blood count, cell count or blood chemistry after FUS.

A veterinarian with extensive experience with NHPs conducted the neurobehavioral examination in NHPs 1 and 2 daily after FUS treatment (Table S7). There were no changes observed in head posture, ambulation, proprioception, fine motor movement, assessed cranial nerve function, or cranial nerve reactions in either animal at any time point. The day after the FUS procedure, the short-term survival NHP 1 showed signs of mild anxiety through an increased reaction to noise in the environment and decreased willingness to come to the front of the cage. This was interpreted as a fearful response. There was no evidence of any neurological abnormality. After 24 h, the animal showed a willingness to come to the front of the cage but was still startled by loud noises. Both NHPs reported a normal score equal to 24 on the quantitative neurological examination each day and were bright, alert, and responsive throughout the examination period.

### NHP cognitive function following FUS-mediated BBB opening

NHPs 3 and 4 were unilaterally treated in the left hemisphere with clinically-relevant FUS parameters, using the FDA-approved Definity microbubble dose (i.e., 10 μl/kg). Contrast-enhanced T_1_-weighted MRI confirmed the BBB opening in the left PFC of both NHPs 3 and 4 (Fig. 1b and Fig. S3). Interestingly, both BBB openings were located in the vicinity of the left arcuate sulcus. This may be due to ultrasound surface effects occurring within the interfaces of the natural folding parts of the NHP brain. Similar effects have also been reported in humans^75^. The BBB opening volume was 415 mm^3^ and 1007 mm^3^ for MI of 0.4 (NHP 3) and 0.8 (NHP 4), respectively.

**Figure 4:**
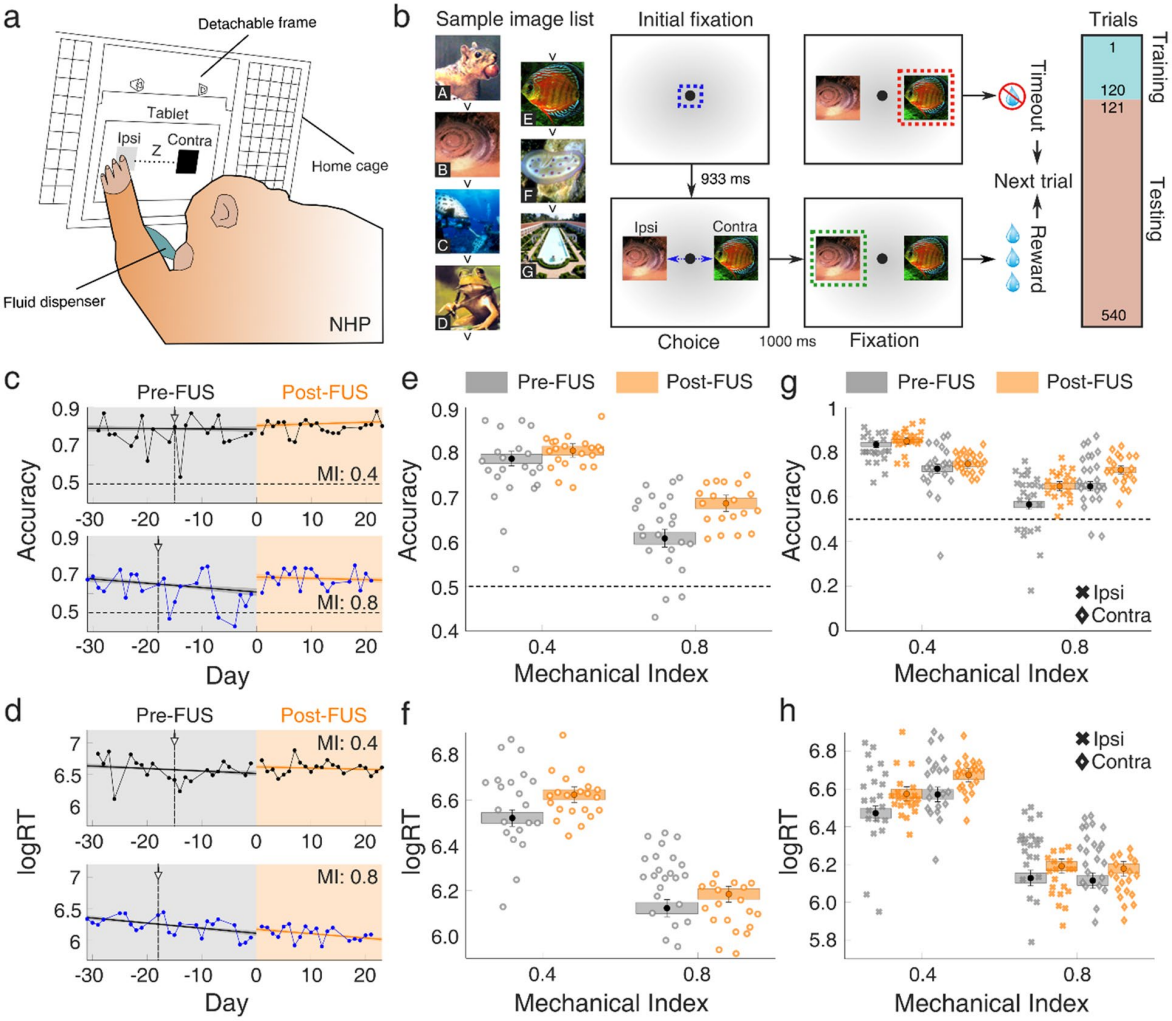
Cognitive function in non-human primates following focused ultrasound treatment. (**a**) Inference test setup for the assessment of accuracy and reaction time in a complex visual task. A tablet fixed within a detachable frame was positioned at the home cage of NHPs 3 and 4 on a daily basis. A set of two images was presented in each trial, one at the left (i.e., ipsilateral to the FUS treatment) side and one at the right (i.e., contralateral to the FUS treatment) side. A fluid dispenser provided water as a reward for each correct answer. (**b**) Inference test design. Two images were randomly selected from a list of 7 images, each carrying a different inference value (**A**–**G** had decreasing implicit value; e.g., picture B had a higher implicit value than picture E etc.), and presented on the screen. The set of images was different for each day. Initial fixation was achieved with a point presented at the monitor for 933 ms. The two images were then presented for 1 s. When NHPs selected the correct answer on time, they were rewarded. In contrast, there was no reward for erroneous responses. The first 120 trials constituted the training phase, while the following 420 trials constituted the testing phase. (**c**) Daily accuracy, for MI of 0.4 (top, NHP 3) and MI of 0.8 (bottom, NHP 4). The mean estimate of the logistic regression for accuracy is plotted with a solid line (black line: pre-FUS; orange line: post-FUS), with shading indicating the 95% confidence interval for the mean. Each data point represents the average accuracy on a given day (n = 540 trials). (**d**) Daily reaction time, for MI of 0.4 (top, NHP 3) and MI of 0.8 (bottom, NHP 4). The mean estimate of the logistic regression for reaction time is plotted with a solid line (black line: pre-FUS; orange line: post-FUS), with shading indicating the 95% confidence interval for the mean. Each data point represents the average reaction time on a given day (n = 540 trials). (**e**) Accuracy intercept on day 0 before (gray boxes) and after (orange boxes) FUS treatment at MI of 0.4 and 0.8. (**f**) Reaction time intercept before (gray boxes) and after (orange boxes) FUS treatment at MI of 0.4 and 0.8. (**g**) Accuracy intercept on day 0 with ipsilateral (crosses) and contralateral (diamonds) targets, before (gray boxes) and after (orange boxes) FUS treatment at MI of 0.4 and 0.8. (**h**) Reaction time intercept on day 0 with ipsilateral (crosses) and contralateral (diamonds) targets, before (gray boxes) and after (orange boxes) FUS treatment at MI of 0.4 and 0.8. Gray and orange areas in (**c**) and (**d**) represent the period before and after FUS application, respectively. Data in (**e**), (**f**), (**g**), and (**h**) are presented as boxplots which correspond to estimated performance on day 0. Whiskers represent the 95% confidence interval, whereas boxes represent the 80% confidence interval. Accuracy and reaction rate for each day were the averages of all completed trials. The vertical line in (**c**) and (**d**) denotes the day of the pre-FUS MRI. Sample images in (**b**) were taken from a “free-to-use” collection of stock photos from a CD-ROM acquired in the 90 s. NHP, non-human primate; FUS, focused ultrasound; Ipsi, ipsilateral (i.e., left) targets; Contra, contralateral (i.e., right) targets; logRT, natural logarithm of reaction time.

Behavioral testing was conducted in the NHP home cage (Fig. 4a), using sets of images with an implicit order that the NHP could learn (Fig. 4b). Accuracy (Fig. 4c) and reaction time (Fig. 4d) fluctuated over time for both NHPs. NHP 3 performed considerably better than NHP 4 in terms of accuracy. Both NHPs had a decrease in the accuracy shortly after the pre-FUS MRI (vertical line with an arrow in Fig. 4c, d), which was reversed within 1–2 days. Such a decrease did not occur after the FUS treatment and the post-FUS MRI scan. Increased accuracy was associated with a slower reaction time for NHP 3.

Logistic regression analysis was performed to assess the step changes in performance immediately after the FUS intervention. Neither subject appeared impaired with respect to response accuracy as a function of FUS; instead, response accuracy slightly increased for both NHPs immediately after FUS (Fig. 4c, e). Reaction times generally showed a moderate decline (i.e., increase in speed) over the course of the experiment. FUS exposure led to slower reaction times (Fig. 4f), followed by a continued decline (Fig. 4d). An examination of ipsilateral vs. contralateral response accuracy (Fig. 4g) and reaction time (Fig. 4h) provided a similar trend, suggesting that ipsilateral performance immediately after FUS was not differentially affected by the procedure compared to contralateral performance. In terms of average performance, overall accuracy increased post-FUS compared to pre-FUS for both NHPs (Table S8). Accuracy increased from 0.76 ± 0.08 pre-FUS to 0.80 ± 0.03 post-FUS for NHP 3, and from 0.62 ± 0.08 to 0.68 ± 0.05 for NHP 4. Interestingly, aside from the higher mean accuracy, the accuracy deviation over time was lower in the post-FUS period. Reaction time on average increased for NHP 3 from 6.57 ± 0.18 to 6.61 ± 0.1, but decreased for NHP 4 from 6.24 ± 0.14 to 6.12 ± 0.1. As seen in the logistic regression model, there was a downward trend of reaction time over time for both the pre-FUS and post-FUS epochs, leading to decreased average reaction time.

There was limited variation between the accuracy with left/ipsilateral targets and right/contralateral targets (Fig. S12) or between training/testing (Fig. S13) over time. Increased average accuracy post-FUS, when compared to pre-FUS, was observed for both ipsilateral and contralateral targets (Fig. 4g). The accuracy increase was higher for the contralateral targets for both NHPs, indicating that there was no functional impairment on the contralateral side of the BBB opening. Reaction times were not affected in NHP 3, but were reduced in NHP 4 for both ipsilateral and contralateral targets.

These findings demonstrate that clinically-relevant FUS is safe in a large animal model and does not lead to deterioration of cognitive performance within the context of the presented case studies (Fig. 4). An overview of the behavioral test outcomes can be found in the Supplementary materials (Table S8).

## Discussion

Noninvasive and targeted BBB disruption (Fig. 1) using acoustically-monitored FUS (Fig. 2) elicits a short-term immune response in the NHP brain (Fig. 3) and does not exacerbate the average NHP performance in a visual-motor learning test (Fig. 4) within our limited sample number. Although FUS-triggered immune response has been the focus of multiple rodent studies^38,39,74,76^, there has been no evidence of such a response in an NHP model, which most closely mimics the human skull and brain structure. Here, we used clinically-relevant parameters and a clinical neuronavigation-guided FUS system prototype^62^, which was approved by the FDA for use in human studies at MI of 0.4 (NCT04118764), based on the data presented herein.

The immune response on day 2 was characterized by increased microglia density in the periphery of brain vessels following BBB opening (Fig. 3). Brain-resident microglia and blood-borne macrophages appeared to migrate towards compromised vessels (Fig. 3e), possibly driven by a concentration gradient of albumin^34,77^. Reactive astrocytes have been previously reported after FUS-induced BBB disruption in rodents^25,41,78^ or after BBB breakdown in pathological conditions^79,80^. The extent of immune response depended on the applied acoustic pressure, or conversely, on the mechanical stresses exerted on the vascular walls. Treatment at high pressure triggered strong perivascular effects (Fig. 3b, c) and substantial microgliosis, with a higher Iba1^+^-CD68^+^ cell density in the perivascular areas 2 days post-FUS, compared to the baseline (Fig. 3e, g). This response was no longer detectable 18 days after treatment (Fig. 3), corroborating previous studies describing its resolution within a few days post-FUS^38–40,74^. In contrast, treatment at low pressure had no effect on the inflammatory cell density at neither the 2-day nor the 18-day time point (Fig. 3g).

DCX is typically expressed in neurogenic areas of the adult brain hippocampus, but DCX^+^ cells have also been reported in other areas, such as the cortex^81,82^. However, DCX may also be expressed by certain glial cells in pathological conditions^73^. FUS-triggered neurogenesis has been attributed to an upregulation of trophic and growth factors, such as brain-derived neurotrophic factor (BDNF), fibroblast growth factor (FGF), and vascular endothelial growth factor (VEGF)^24,38,47^. Upregulation of the latter can stimulate vessel growth and angiogenesis following FUS exposure^41^, potentially leading to increased blood flow through the cortical vasculature. Additionally, targeted and localized BBB opening alters neurovascular coupling^44^ and resting-state functional connectivity^45^. Based on our observations in NHPs, futures studies should further explore the connection between immune response and immature neurons or neuronal excitability in rodents, coupled with functional modalities such as functional MRI and electrophysiology.

FUS-triggered immune response and neurogenesis have been correlated with improved cognitive performance of a transgenic mouse model of AD^24,34^. We show here that BBB opening does not compromise cognitive function in healthy non-impaired NHPs (Fig. 4), which are the closest model to humans. Future work should investigate whether a similar safety profile is observed in geriatric subjects, whose brain may have different properties compared to younger healthy adults**.**

We hypothesize that the observed increase in immature neurons (Fig. 3) may be correlated with the higher response accuracy following FUS (Fig. 4). This hypothesis can be tested in rodent studies, for example by genetically ablating neurogenesis at specified time points^83^. We did not perform NeuN staining to establish that neurogenesis has been completed and has led to increased density of mature neurons within the treated areas at either time point. Formation of new neurons and enhanced neuronal excitability due to the immune response^43^ within the prefrontal and motor cortices may have partially contributed to the increased response accuracy of NHPs post-FUS at the examined time scales (Fig. 4, Figs. S12 and S13). However, this remains a hypothesis and should be evaluated in future studies.

The behavioral outcomes described here generally agree with recently published studies, showing an accuracy increase of NHPs during and immediately after FUS treatment^30,54,55^. In this study, we found that a single FUS treatment does not have a negative impact on the NHP performance during complex cognitive tasks. Interestingly, accuracy was higher in targets appearing at the contralateral visual field (Fig. 2g, h, Table S8), suggesting that BBB opening at the ipsilateral side may be a likely cause of the observed effect. The results of the current and previous studies demonstrate that FUS-mediated BBB opening at clinically relevant parameters is histologically safe and does not compromise cognitive function within the context of a visual-motor task.

The volume of BBB disruption (Fig. 1c) correlated well with harmonic stable cavitation dose (Fig. 2h), as shown before^64,70,84^. FUS therapies can be performed using real-time closed-loop systems^85–87^, to adjust the BBB opening volume and, consequently, the extent of the immune response (Fig. 3) based on acoustic feedback (Fig. 2). Furthermore, passive acoustic mapping in either the time^88–93^ or frequency^94–96^ domain can predict the location of the induced BBB opening within the brain. FUS treatments with clinically relevant parameters had an acceptable radiological safety profile (Fig. 1e, f, Figs. S4, S5) and produced BBB disruption that lasted for 3 days (Fig. 1g). The ability to control treatment progression in real-time is a unique feature that can be employed for localized immunotherapies or targeted drug delivery into the brain. Preliminary clinical studies of FUS-induced BBB opening in AD patients suggest a potential reduction in the amyloid load of the treated areas compared to contralateral areas^97^, but there has been no measurable change in cognitive function to date^31^. However, this may be due to the limited size of BBB disruption in these early feasibility studies or the pathology itself. Ongoing clinical trials investigate the effects of FUS-induced BBB openings in patients with AD^31^, glioblastoma multiforme^63,75,98^, and amyotrophic lateral sclerosis^99^.

The current study has a number of important limitations. Neurogenesis and immune response are potential contributors to cognitive performance following FUS treatments. However, they are likely a small subset of the downstream effects triggered by FUS exposure and BBB opening^100^, which are currently not fully understood. Behavior-related brain circuitry may be affected following targeted BBB disruption by a plethora of factors, such as cortical blood oxygenation levels, cerebral blood flow, SSEP modulation or synchronization, neurovascular coupling, and structural or functional connectivity reorganization^44,51,56–58,101^. Due to the case-study design and nature of NHP experiments, we could not incorporate functional measurements and sham exposures in this work. Future studies in wild-type rodents should focus on identifying the contribution of each of these effects in cognitive function across different temporal and spatial scales, and include comparisons with sham animals, to complement the within-subject comparison done here.

Additionally, the NHPs in our study were young adults (7–8 years old), whose brains may have higher neuroregenerative capacity and different mechanical properties compared to geriatric patients. NHPs were exposed to a single FUS session since our purpose was to identify the effects triggered by a single localized BBB opening in FUS-naïve brains. This experimental design imitated our imminent phase I clinical trial with AD patients (NCT04118764), which will involve one FUS session per participant, and was developed in coordination with the CDRH of the FDA to support translation to humans. Clinical applications may require multiple treatment sessions, which will likely have an accumulating effect on the brain tissue. The requirement for a single FUS session in FUS-naïve animals necessitated the study restriction of n = 4 animals and n = 6 total sonications. As a consequence, several aspects of this study necessarily included a single data point per condition in lieu of a case study, e.g. for the BBB closing timeline at low and high pressure. The BBB closing timeline presented here assumed that the BBB closing process was uniform across animals. However, in this case study, only one animal was scanned on day 1 and day 3, to derive the percentage of the BBB remaining open following FUS in both hemispheres. In this way, the variability across animals in each time point could not be addressed. Thus, we could not perform a meaningful statistical analysis. Similarly, this study was not time-continuous within the same animal. Therefore, our analysis assumed that the initial histological effects would be similar between NHP 1 and NHP 2. Future rodent studies should employ multiple animals, to ensure the reproducibility of the observations reported herein across species and animals. Regardless, our preliminary findings in this limited NHP sample number support that neuronavigation-guided FUS with a single-element FUS transducer is a non-invasive method capable of reversibly opening the BBB, without substantial histological or behavioral impact with potential, albeit slight, cogntive improvement in an animal model closely resembling humans.

## Supplementary Information

Supplementary Information.

## Data Availability

All data associated with this study are present in the paper or the supplementary materials. All data are available upon request from the corresponding author (E.E.K., ek2191@columbia.edu).
