# Supplementary material for: Safety evaluation of a clinical focused ultrasound system for neuronavigation guided blood-brain barrier opening in non-human primates
Source: Sci Rep. 2021 Jul 22;11:15043. doi: 10.1038/s41598-021-94188-3 (PMC8298475; doi:10.1038/s41598-021-94188-3)
Supplement: Supplementary file 1 — Supplementary Information. [file 41598_2021_94188_MOESM1_ESM.docx]

**Safety evaluation of a clinical focused ultrasound system for neuronavigation guided blood-brain barrier opening in non-human primates**

**Supplementary material**

**__________________**

**Antonios N. Pouliopoulos^1^, Nancy Kwon^1^, Greg Jensen,^2,3^ Anna Meaney^4^, Yusuke Niimi^1^, Mark T. Burgess^1^, Robin Ji^1^, Alicia J. McLuckie^5^, Fabian Munoz^4^, Hermes A.S. Kamimura^1^, Andrew F. Teich^6^, Vincent P. Ferrera^2,4,7^, and Elisa E. Konofagou^1,8^**

1. Department of Biomedical Engineering, Columbia University, New York City, NY 10032, USA
2. Department of Neuroscience, Columbia University, New York City, NY 10032, USA
3. Department of Psychology, Columbia University, New York City, NY 10032, USA
4. Mortimer B. Zuckerman Mind Brain Behavior Institute, Columbia University, New York City, NY 10027, USA
5. Institute of Comparative Medicine, Columbia University, New York City, NY 10032, USA
6. Department of Pathology and Cell Biology, Columbia University, New York City, NY 10032, USA
7. Department of Psychiatry, Columbia University, New York City, NY 100032, USA
8. Department of Radiology, Columbia University, New York City, NY 10032, USA

**Contents**

**Page**

| Supplementary methods | | | | | 3 | |
| --- | --- | --- | --- | --- | --- | --- |
| Supplementary results | | | | | 10 | |
| Fig. S1. NHP and clinical setup for non-invasive blood-brain barrier opening | | | | | 11 | |
| Fig. S2. Targeting planning and accuracy using the Brainsight neuronavigation system | | | | | 12 | |
| Fig. S3. Axial slices of T_1_-weighted MRI scans after focused ultrasound treatment | | | | | 13 | |
| Fig. S4. Axial slices of T_2_-weighted MRI scans after focused ultrasound treatment | | | | | 14 | |
| Fig. S5. Axial slices of susceptibility-weighted MRI scans after focused ultrasound treatment | | | | | 15 | |
| Fig. S6. Spectral analysis and cavitation levels | | | | | 16 | |
| Fig. S7. Iba1/CD68 quantification | | | | | 17 | |
| Fig. S8. Doublecortin quantification | | | | | 18 | |
| Fig. S9. Axonal and neurofilament morphology after focused ultrasound treatment | | | 19 | | | |
| Fig. S10. Focused ultrasound-triggered apoptosis | | | 20 | | | |
| Fig. S11. Caveolar endocytosis is promoted following focused ultrasound treatment | 20 | | | | | |
| Fig. S12. Accuracy and reaction time per target location | 21 | | | | | |
| Fig. S13. Accuracy and reaction time per phase | 22 | | | | | |
| Fig. S14. Blood-brain barrier opening quantification algorithm and graphics user interface | 23 | | | | | |
| Table S1. Complete blood count 2 days post-treatment | 24 | | | | | |
| Table S2. Complete blood count 18 days post-treatment | 25 | | | | | |
| Table S3. Cell count 2 days post-treatment | 26 | | | | | |
| Table S4. Cell count 18 days post-treatment | | 26 | | | | |
| Table S5. Comprehensive chemistry panel 2 days post-treatment | | | | 27 | | |
| Table S6. Comprehensive chemistry panel 18 days post-treatment | | | | | | 28 |
| Table S7. Neurological examination for assessment of focused ultrasound neurological safety | | | | | | 29 |
| Table S8. Cognitive performance overview | | | | | | 30 |
| References | | | | | | 31 |

**Supplementary methods**

*Focused ultrasound setup*

The non-human primate (NHP) focused ultrasound (FUS) setup (Figs. 1a and S1a) was identical to the neuronavigation-guided clinical-prototype FUS system (Fig. S1b), which has been described in detail elsewhere.^1^ The 0.25-MHz FUS transducer was driven by a function generator (33500B Series; Agilent technologies, Santa Clara, CA, USA) through a 55 dB RF power amplifier (A150; E&I, Rochester, NY, USA) using clinically-relevant parameters. The amplified signal was fed through an impedance matching box to match the impedance of the FUS transducer with the 50 Ω emission system. A water degassing system (WDS105+; Sonic Concepts, Bothell, WA, USA) was used to fill the transducer cone with degassed water and inflate or deflate the cone according to the treated location. Reflective beads were attached to the transducer to enable real-time tracking of its location through an infrared camera acting as a position sensor and neuronavigation guidance (BrainSight; Rogue Research, Montreal, QC, Canada). Both subject and tool trackers were registered in real space before the experiment.

Microbubble emissions emanating from within the vasculature (Fig. 2a – left) were detected using a 1.5 MHz passive cavitation detector (diameter: 32 mm, focal depth: 114 mm; ndtXducer, Northborough, MA, USA). Signal was received through a pulser/receiver (5077PR; Olympus, Waltham, MA, USA), amplified by 30 dB, and recorded through a GaGe digital oscilloscope (1250X, 12-bit A/D resolution; DynamicSignals LLC, Lockport, IL, USA). The sampling frequency was 50 MSa/s and the total acquisition length was 10ms (Fig. 2a - middle). Passive cavitation detection (PCD) provides information about the acoustic cavitation magnitude, duration, and mode within the focal volume.^2–7^ Using an array of receivers one can localize the acoustic sources and produce maps of cavitation activity.^8–11^ Single-element PCD was used here to characterize the microbubble behavior within the vasculature.

*Acoustic cavitation analysis*

We computed the harmonic stable (SCD_h_), ultraharmonic stable (SCD_u_), and inertial (ICD) cavitation dose, as described before.^12,13^ Time-domain signals (Fig. 2a – middle) were transformed into the frequency domain through a fast Fourier Transform (segment size: 524,288 data points), performed in MATLAB (The Mathworks, Natick, MA, USA). Spectra were normalized to their maximum amplitude and presented in a logarithmic (dB) scale (Fig. 2a – right). Spectrograms were constructed by stacking spectra from all 240 consecutive pulses. A 5×5-pixel Gaussian filter was applied in the 2D spectrograms, in order to smooth noisy spectral features.

We then isolated three spectral domains to calculate the relevant cavitation levels or doses (Fig. 2a – right). Harmonic peaks were multiple integers of the fundamental frequency, i.e. $f_{h,n}=nf_{0}$ (green areas); ultraharmonic peaks were found as $f_{u,n}=(n+1/2)f_{0}$ (blue areas); and, broadband emissions were all intermediate spectral areas $f_{b}$, with $f_{h,n}+10kHz<f_{b}<f_{u,n}-10kHz$ and $f_{u,n}+10kHz<f_{b}<f_{h,n+1}-10kHz$ (red areas). $f_{0}$ was the center frequency of the therapeutic beam, equal to 0.25 MHz, while $n$ was the harmonic number ranging from 3 to 10. The fundamental frequency and second harmonic were not included in the cavitation dose calculation, because of large reflections on the NHP skull at these frequencies.

Cavitation levels were calculated as the root-mean-square (RMS) of the FFT amplitude in each frequency domain and for each therapeutic pulse: , $dSCD_{u}=\sqrt{\left\langle\left|FFT\right|_{f_{u,n}}^{2}\right\rangle_{n}}$ and $dICD=\sqrt{\left\langle\left|FFT\right|_{f_{b}}^{2}\right\rangle}$. The sum of cavitation levels throughout the $T=2\min$ FUS treatment provided the cavitation dose per treatment: $SCD_{h}=\sum_{t=0}^{T} dSCD_{h,t}$, $SCD_{u}=\sum_{t=0}^{T} dSCD_{u,t}$and $ICD=\sum_{t=0}^{T} dICD_{t}$. The total cavitation dose was the sum of all three cavitation doses: $CD=SCD_{h}+SCD_{u}+ICD$.

*MRI sequences*

BBB opening was assessed as described before,^14^ approximately 2 hours after FUS treatment with T_1_-weighted MRI (3-D Spoiled Gradient-Echo, TR/TE = 20/1.4 ms; flip angle: 30°; NEX = 2; spatial resolution: 500 × 500 µm^2^; slice thickness: 1 mm with no inter-slice gap). T_1_-weighted scans were acquired before (1 hour post-FUS) and 50 min after IV administration of 0.2 ml/kg gadodiamide MRI contrast agent (Omniscan; GE Healthcare, Bronx, NY, USA), which is normally impermeable to the BBB (molecular weight: 591.7 Da). BBB opening was quantified by comparing the pre- and post-contrast administration T_1_ scans (Fig. S14). Safety outcomes were assessed with axial T_2_-weighted MRI (TR/TE = 3000/80 ms; flip angle: 90°; NEX = 3; spatial resolution: 400 × 400 µm^2^; slice thickness: 2 mm with no inter-slice gap) and susceptibility-weighted imaging (SWI; TR/TE = 19/27 ms; flip angle: 15°; NEX = 1; spatial resolution: 400 × 400 µm^2^; slice thickness: 1 mm with no inter-slice gap). Pre-contrast T_1_-weighted, T_2_-weighted and SWI sequences were acquired approximately 1 hour after the FUS treatment and prior to Omniscan IV administration. MRI scans were performed using the 3T MRI scanner at Columbia University Medical Center (NHPs 1 and 2) and the 3T MRI scanner in the Jerome L. Greene Science Center at the Columbia Zuckerman Institute for Neuroscience (NHPs 3 and 4).

*Neurobehavioral examinations*

Observational neurological examinations were performed daily by a veterinarian experienced with NHPs, based on a quantitative scoring system (Table S7). Examinations were performed either in the animal’s home cage or adjacent to their home cage in an attached and familiar play cage. Initially the animal was observed for 1-2 minutes to assess mentation and posture. Enrichment food items were then offered in multiple positions to encourage head and eye movement, ambulation of all four limbs, and fine motor movements. This allowed assessment and semi-quantitative grading of visual tracking (Cranial nerves [CN] III, IV, and VI), pupil size and reflex (CN II and III), ambulation and proprioception, and fine motor movements. Facial muscle movement and tone (CN VII) was also assessed during this time, and whilst enrichment was fed. These values were evaluated on a scale of 0 (absent), 1 (depressed), 2 (normal), or 3 (hyper-reactive), on both the right and left sides. Appetite and interest in enrichment was also assessed. Any abnormalities during the examination or during daily health checks were noted.

*NHP euthanasia*

NHPs 1 and 2 were euthanized to allow for histological analysis of the bilaterally treated brains. NHP 1 was euthanized on day 2 post-FUS, in order to assess short-term effects of the FUS treatment. NHP2 was euthanized on day 18 post-FUS, in order to assess long-term effects of the focused ultrasound treatment. NHPs were perfused transcardially with a mixture of phosphate buffered saline (PBS) and heparin to extract blood from the vasculature, followed by fixation with 4% paraformaldehyde (PFA). The animal was initially sedated with ketamine and dexmedetomidine in order to be intubated for isoflurane gas administration and for placement of peripheral intravenous (IV) catheter. The animal was maintained in deep anesthesia through inhalable isoflurane (2-3%) during the procedure. The deep plane of anesthesia was continuously confirmed by the lack of palpebral reflex, and lack of withdrawal on toe pinch reflex prior to incision.

The chest was shaved to prepare the thorax for surgery and scrubbed with chlorhexidine followed by betadine to disinfect the skin. A thoracotomy was performed and an incision was made in the pericardium to expose the heart. An IV line was inserted into the left ventricle and clamped in place. The descending aorta was clamped and a drainage incision was made in the right atrium. Death was confirmed by lack of heartbeat and respiration. Craniotomy was then performed to obtain brain tissue for analysis. Brain tissues were stored in 4% PFA for a period of 2-3 weeks. They were then processed by the histology core and immunohistochemistry core housed within the Herbert Irving Comprehensive Cancer Center of Columbia University.

*Immunohistochemistry – Iba1-CD68 and GFAP*

Paraffin-embedded NHP brain sections were first stained for Iba1-CD68 to identify microglia presence and functional state. Antigen retrieval was performed with a low pH target retrieval solution (S236984-2, citrate pH 6; Agilent Dako) for 20 minutes in a steamer and then cooled for 20 minutes. Sections were incubated with goat anti-Iba1 (ab5076, dilution 1:500; abcam) and rabbit anti-CD68 (ab125212, dilution 1:300; abcam) primary antibodies at room temperature (RT) for 1.5 hours. Samples were then incubated with the respective secondary antibodies, i.e. rabbit anti-goat IgG antibody (ab6697, dilution 1:200; abcam) and goat anti-rabbit IgG antibody (ab6702, dilution 1:200; abcam), for 30 minutes at RT. Sections were developed using 3,3’-diaminobenzidine (DAB), until visible colored tissue could be observed.

Paraffin-embedded NHP brain sections were then stained for GFAP to identify astrocyte presence and reactivity. Antigen retrieval was performed using a low pH target retrieval solution (S236984-2, citrate pH 6; Agilent Dako) heated for 10 minutes in a microwave and cooled down for 30 minutes. Sections were incubated with rabbit anti-GFAP antibody (ab16997, dilution 1:300; abcam) for 30 minutes at RT. They were then incubated with the secondary goat anti-rabbit IgG antibody (ab205718, dilution 1:200; abcam) for 30 minutes at RT. This was followed by incubation with Avidin Biotin Complex (ABC) (PK-7200 ABC kit, dilution 1:50; Vector laboratories) for 30 minutes followed by color development using DAB.

To quantify the immune response, we estimated the average density of microglia within the BBB opening area (Fig. S7). Due to the spectral overlap of the Iba1 and CD68 chromogens, we converted bright field images into grayscale and thresholded to identify monocyte lineage cells. Both Iba1 and CD68 are typically used to identify microglia; however, they do not always overlap, with Iba1 being a more suitable marker for morphology estimation in the absence of pathology and CD68 reflecting an immune activation and response to tissue damage.^15^ Therefore, both Iba1^+^ and CD68^+^ cells were taken into account to identify microglia and/or macrophages.

*Immunohistochemistry – DCX and Cav-1*

Paraffin-embedded NHP brain sections were also stained for doublecortin (DCX), and caveolin-1 (Cav1). Brain samples were deparaffinized and hydrated in day 1. Sections were first embedded in xylene for 5-10 min (x 2 times). Progressively lower concentrations of ethanol were then applied: 100% ethanol (5 min); 100% ethanol (2 min); 95% ethanol (2 min); 85% ethanol (2 min); 70% ethanol (2 min); 50% ethanol (2 min). Finally, the sections were hydrated with deionized water (5 min, x 2 times). Antigen retrieval was performed using 100% formic acid for 5 min, followed by washing with deionized water (5 min, x 3 times). The blocking step was conducted with 5% normal goat serum in PBS – 0.3% triton-X (HFH10, Thermo Fisher Scientific) solution (PBSt) for 30 min.

Primary antibodies were diluted in 1% normal goat serum (NGS) with 0.3% PBSt. Sections were incubated with either goat anti-doublecortin (ab113435, dilution 1:500; abcam) or rabbit anti-caveolin-1 (cell signaling 3267, dilution 1:400; cell signaling technology), either in a humid 4 ^o^C chamber or at RT overnight. In day 2, slides were washed 3 times with PBSt for 10 min. DCX-stained samples were counter-stained with rabbit anti-GFAP (ab16997, dilution 1:500; abcam) to detect reactive astrocytes. Sections were incubated with the secondary antibody goat anti-rabbit Alexa 488 (ab150077, dilution 1:500; abcam) and, for the DCX-stained sections, also with rabbit anti-goat Alexa 647 (ab150143, dilution 1:500; abcam). Samples were stored in darkness within a humid chamber or at RT for 3 hours. The secondary antibodies were previously diluted in 1% NGS with 0.3% PBSt. Sections were then washed 3 times with PBSt for 10 minutes. Finally, we added mounting solution with DAPI (P36971; Thermo Fisher Scientific) and a coverslip prior to fluorescence imaging.

*Iba1^+^-CD68^+^ and DCX^+^ cell counting*

Iba1^+^-CD68^+^-stained samples were imaged at 10x magnification in bright field using an upright microscope (Leica DM6 B; Leica Microsystems Inc., Buffalo Grove, IL, USA). Perivascular areas within and outside the focal areas were randomly selected (n=5 per hemisphere and time point). Areas not exposed to FUS were used to derive the baseline microglia density. Raw images were opened with ImageJ^16^ (Fig. S7a) and were converted to 8-bit grayscale (Fig. S7b). An arbitrary threshold was applied to remove the background and isolate Iba1^+^-CD68^+^ signal (Fig. S7c). The total area of pixels with intensity above the specified threshold was calculated. Next, 10 isolated microglia without processes were selected and their average size was computed (Fig. S7d). The average area per Iba1^+^-CD68^+^ cell was 65 ± 33 μm^2^. To estimate the glial cell density in each image, the total area occupied by Iba1^+^-CD68^+^ cells was divided by the average cell size, and then normalized to number of Iba1^+^-CD68^+^ cells per mm^2^.

DCX-stained samples were imaged at 20x magnification in fluorescence mode (red channel-Y5 filter, green channel-GFP filter and blue channel-DAPI filter) using an upright microscope (Leica DM6 B; Leica Microsystems Inc., Buffalo Grove, IL, USA). Images were processed in ImageJ ^16^. Images were decomposed to individual channels, i.e. red for DCX (Fig. S8a), green for GFAP (Fig. S8b) and blue for DAPI (Fig. S8c). Merged images (Fig. S8d) helped identify regions including both DCX^+^ and GFAP^+^ cells. An arbitrary threshold was applied to isolate DCX^+^ cells from the background (Fig. S8e). DCX^+^ cells were identified using the “Analyze particles” function (size: 30 – 1000, circularity: 0 – 1) and an ellipse was assigned to each cell (Fig. S8f). The number of DCX^+^ cells were recorded at random regions of interest within and outside the treated areas (n=5 per hemisphere and time point), based on the contrast-enhanced T_1_-weighted MRI scan. DCX^+^ cells were detected in the proximity of neurogenic areas, e.g. the hippocampus, but also in other cortical and sub-cortical areas that underwent BBB opening, albeit at a lower incidence. Areas not exposed to FUS were used to derive the baseline DCX^+^ cell density, which was non-zero possibly due to unspecific staining or random fluorescence intensity above the specified threshold. Adult neurogenesis occurs throughout life, so a number of DCX^+^ cells were expected in areas such as the sub-granular zone of the dentate gyrus and the sub-ventricular zone of the lateral ventricles. Here, we show that FUS-induced BBB opening increases the density of DCX^+^ cells in these areas and others, confirming previous studies in rodents.^17,18^

The reported Iba1^+^-CD68^+^ and DCX^+^ cell densities were calculated for comparative reasons in randomly selected ROIs where signal was detected, and do not represent the average cell density within the entire treated and non-treated areas. A limitation of the current study is the absence of histological data from control NHPs who were not treated with FUS, due to the nature and cost of experiments with NHPs.

*Blood test analysis*

3 ml of blood were drawn from the IV catheter that was placed for the pre-treatment MRI or euthanasia procedures (day 2 for NHP 1 and day 18 for NHP 2). We performed a single blood draw for each NHP after treatment, so the results should be interpreted within the context of each case study. The procedure was performed by a trained veterinarian. The collected samples were sealed in a sterile container for laboratory testing and were stored in a refrigerator (3-5 ^o^C) until processing. Samples were analyzed for complete blood count, cell count, and comprehensive chemistry panel, and were compared against the baseline to detect any changes related to the FUS procedure.

*Blood-brain barrier Opening Quantification and Analysis (BOQA) graphics user interface (GUI)*

Quantification of the BBB opening across NHPs was performed using an automated image processing algorithm (Fig. S14). In brief, the post-FUS T_1_-weighted scan without contrast was subtracted from the contrast-enhanced T_1_-weighted scan, to produce a difference image. An arbitrary threshold was set to eliminate the background noise and delineate areas with increased contrast. A region of interest (ROI) was defined within the left or right brain hemisphere, to allow for vessel separation, and all pixels with intensity above the specified threshold were overlaid to the T_1_-weighted scan without contrast. To facilitate visualization, pixels with similar intensities were connected with contours. Further adaptation of the threshold was performed to eliminate the noise and areas with marginal intensity increase. The final BBB opening contour surface was used to calculate the BBB opening area per slice, and the BBB opening volume by multiplying the surface area with the slice thickness. The same process was repeated for every slice of the T_1_-weighted scan to calculate the total BBB opening volume throughout the NHP brain (Fig. 1c).

**Supplementary results**

*Training vs. testing phase accuracy and reaction times*

In each behavioral assessment, NHPs were presented with a new set of images. Therefore, the first 120 trials were considered a training phase (Fig. 1b). The remaining 420 trials were considered the testing phase. Accuracy of NHP 3 was either at or higher than chance during training phase (Fig. S13a), while the training accuracy of NHP 4 was at chance, both before and after the FUS treatment (Fig. S13b). On average, NHP 3 had higher accuracy during the training phase post-treatment compared to pre-treatment (Fig. S13c). NHP 4 had marginally lower accuracy during training post-FUS. Testing accuracy was consistently above chance for both NHPs. During the testing phase, NHP 3 had high accuracy, reaching 0.95 on specific days (Fig. S13a). Testing phase accuracy increased after the FUS treatment for NHP 3. and NHP 4, showing that there was no impairment in the inference capacity of the treated NHPs post-FUS. In terms of reaction time (Figs. S13d and S13e), NHP 3 responded slower during the training phase post-FUS, both on day 0 and on average, and marginally faster in testing phase on average. Interestingly, NHP 4 provided faster responses on average during both training and testing phases after treatment. In conclusion, there was no negative impact of the FUS treatment on the accuracy or reaction time in neither the training nor the testing phase.

*Bielschowsky’s silver stain, TUNEL, and Caveolin-1-mediated uptake*

We detected evidence of axonal and cell body injury in the silver stain within the impacted area of the right hemisphere at the 2-day time point (Fig. S9). Similarly, TUNEL^+^ cells indicated apoptosis triggered by FUS exposure at high MI (Fig. S10). These effects were constrained within the perivascular areas exposed to ultrasound at MI of 0.8, and were not present in the left hemisphere at the 2-day time point or in either hemisphere at the 18-day time point. Caveolin-1 appeared to be upregulated within the endothelial cells surrounding arterioles and capillaries at the 2-day time point after treatment at MI of 0.4 (Fig. S11), indicating that caveolin-mediated endocytosis may be a potential mechanism of uptake following low-pressure FUS treatment.

**Supplementary figures**

**
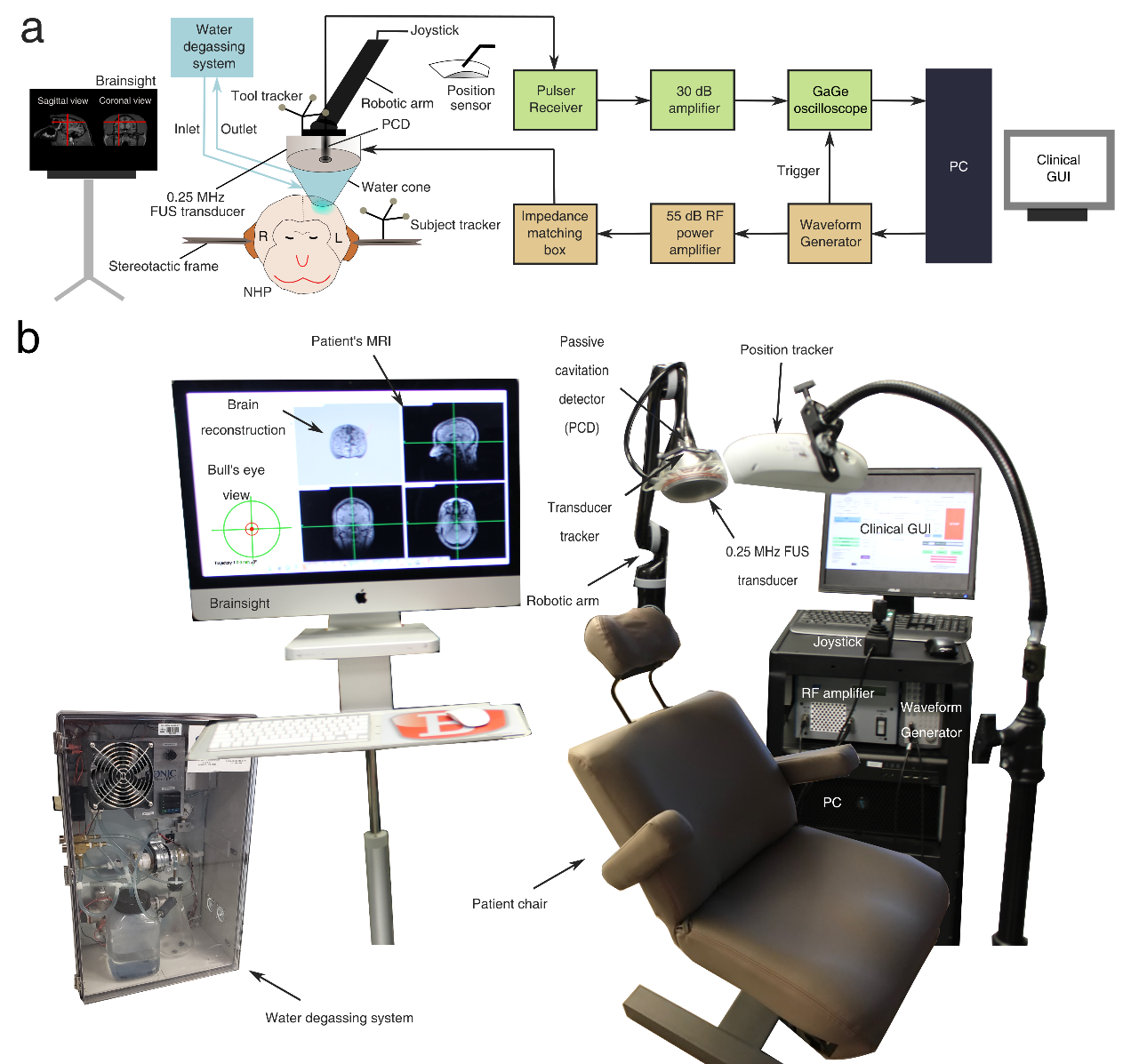
**

**Fig. S1. Non-human primate and clinical setup for non-invasive blood-brain barrier opening.** (**a**) Neuronavigation-guided FUS system for BBB opening in NHPs. The anesthetized NHP was shaved and positioned into a stereotaxic frame. FUS emission (orange boxes) and reception (green boxes) processes were controlled via a PC though a purpose-built GUI. A waveform generator produced 0.25-MHz sinusoidal pulses with length of 2,500 cycles (or 10 ms). The pulses were amplified with a 55 dB power amplifier and were then applied to the 0.25-MHz single-element FUS transducer via an impedance matching box. The FUS transducer was fixed onto a robotic arm, which was manually controlled with a joystick. A water cone was adjusted onto the transducer surface to allow for ultrasound propagation into the NHP skull. The water cone was equipped with inlet and outlet ports for water circulation through a water degassing system. Ultrasound gel was applied on the skin region right below the water cone to minimize ultrasound attenuation. The FUS construct movement was guided by the BrainSight neuronavigation system, which was pre-loaded with the treatment planning T_1_-weighted MRI scan of the treated NHP. The scan included fiducial markers for registration purposes. An infrared position tracker detected the position of the subject tracker and tool tracker in real-time. During FUS treatment, microbubble acoustic emissions were recorded with a 1.5-MHz single element PCD. The signal was captured and amplified by 20 dB via a pulser/receiver operating in receive mode. It was finally digitized and recorded in the PC with a GaGe oscilloscope card. (**b**) Photograph of the clinical neuronavigation-guided FUS setup ^1^ illustrating the equipment described above. Abbreviations. FUS: focused ultrasound; PCD: passive cavitation detector; NHP: non-human primate; BBB: blood-brain barrier; RF: radiofrequency; dB: decibel; GUI: graphics user interface.


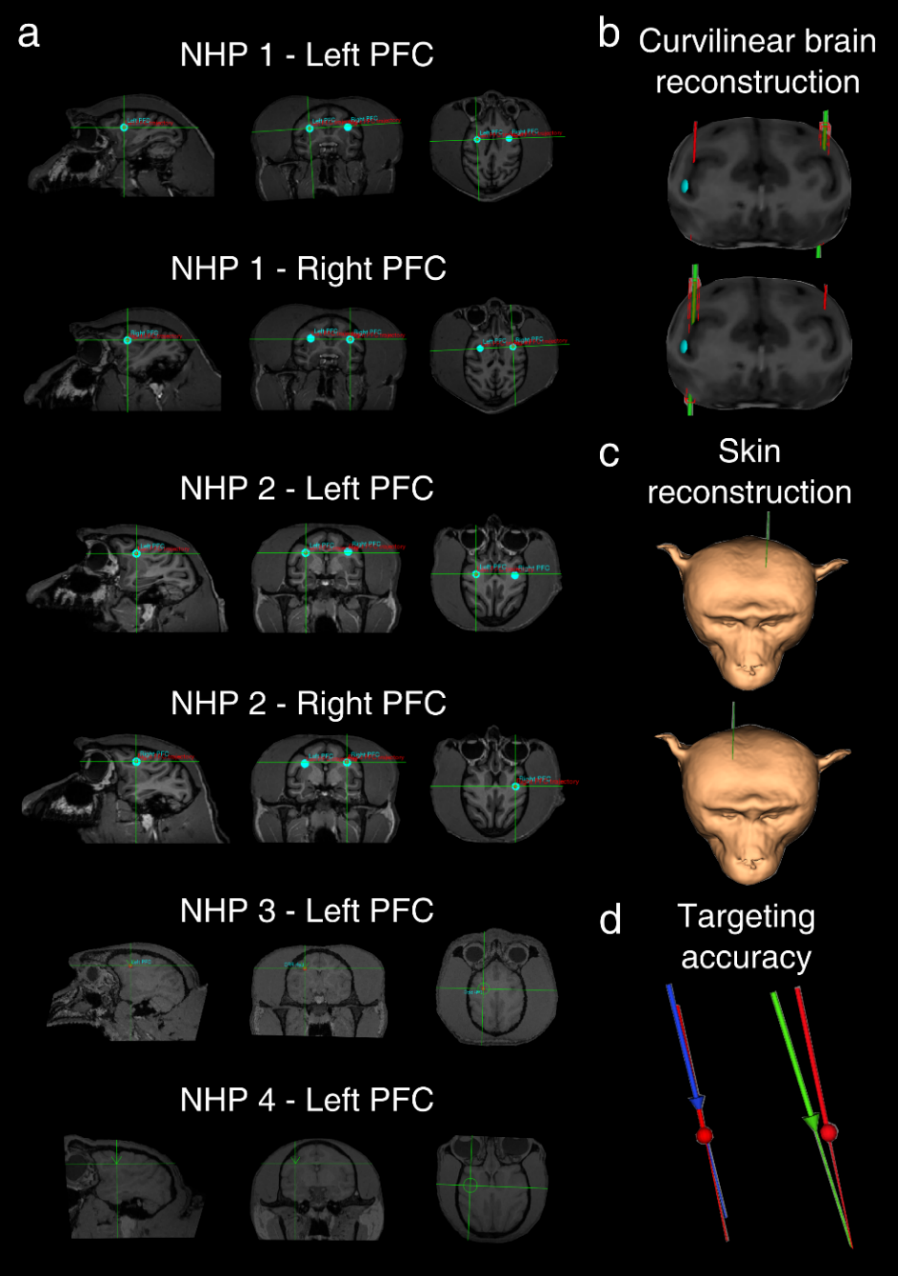


**Fig. S2. Targeting planning and accuracy using the Brainsight neuronavigation system.** (**a**) Targeting planning for NHPs 1, 2, 3, and 4. The targets were selected to be within the PFC. The requirements for the targeting location were: i) part of the PFC with sufficient gray matter volume, ii) targeting two symmetric sites without overlap for MI of 0.4 and 0.8, iii) avoid direct exposure of eyes, iv) avoid oblique incidence angles with respect to skull to minimize attenuation/reflection of the ultrasound beam,^1,19^ v) flat skin access for minimal distortion of ultrasound propagation, vi) target structure that inflicts a behavioral change if damaged. An area fulfilling most of these criteria was the dorsolateral PFC, specifically the 6DC, 6VC, and 8A areas (coordinates: interaural 21 ± 3 mm, bregma: -0.9 ± 3 mm, lateral distance from sagittal midbrain plane: 15 mm). Given the PFC size in rhesus macaques and the size of the focal volume (6 mm × 6 mm × 49 mm), other posterior areas (such as the pre-motor and motor cortices) were within the targeted area. This was preferred over direct exposure of the eyes. NHPs 1 and 2 were treated bilaterally (MI of 0.4 on the left side and MI of 0.8 on the right side). NHPs 3 and 4 were treated unilaterally on the left side at MI of 0.4 and 0.8, respectively. (**b**) Curvilinear brain reconstruction showing the FUS beam planned trajectory with respect to the brain. (**c**) Skin reconstruction showing the FUS beam trajectory with respect to the NHP head. (**d**) Targeting accuracy. Deviation between planned (red lines/spheres) and achieved (blue and green lines/arrowheads) trajectories, illustrating the limitation of the 4-degree-of-freedom robotic arm**.** Brain and skin reconstructions in Fig. S2b and S2c were created with BrainSight software 2.4 (https://www.rogue-research.com). Abbreviations. NHP: non-human primate, PFC: prefrontal cortex; MI: mechanical index; FUS: focused ultrasound.

***
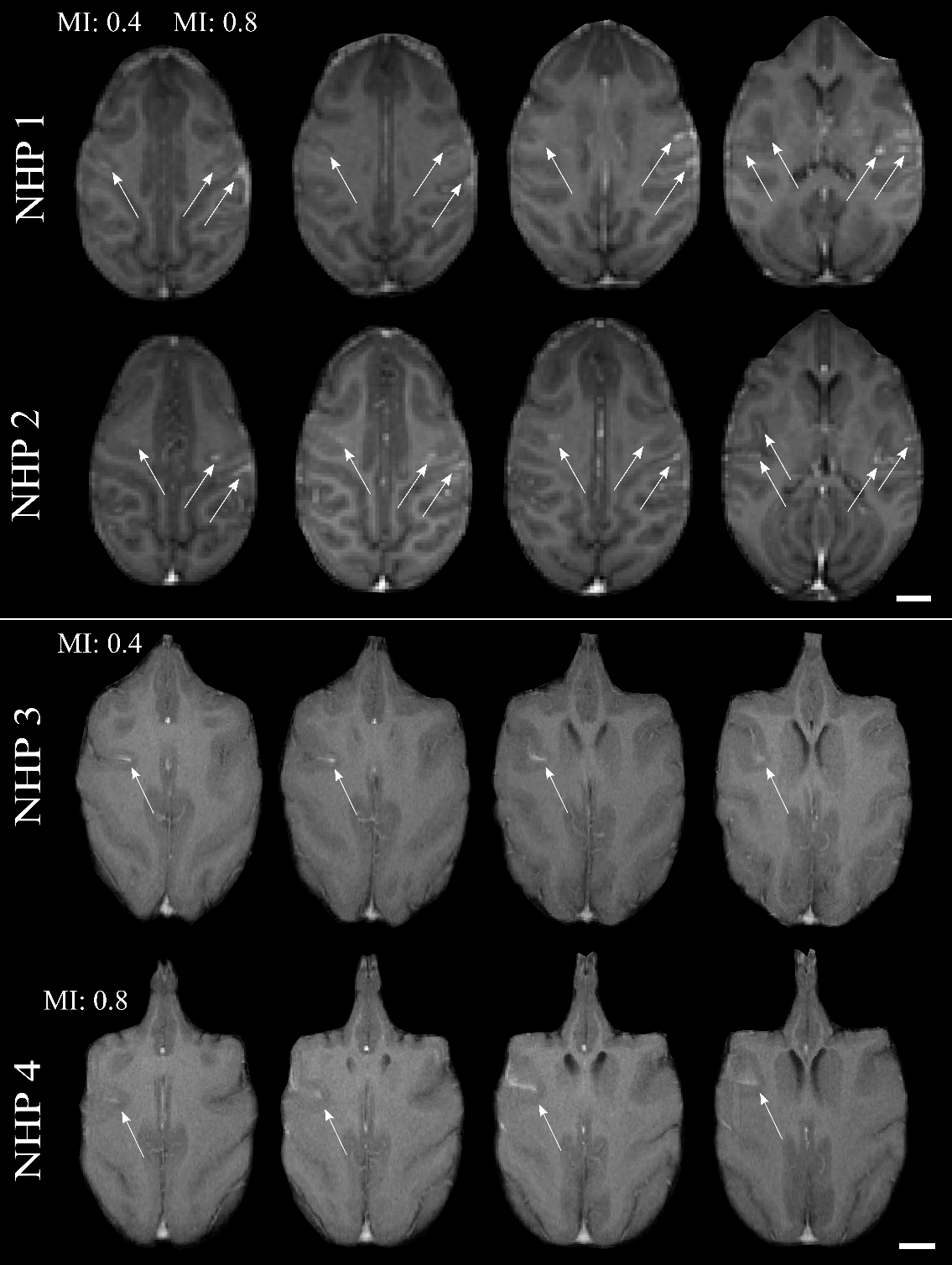
***

**Fig. S3. Axial slices of T_1_-weighted MRI scans after focused ultrasound treatment.** Contrast-enhanced T_1_-weighted MRI was acquired approximately 2 hours after FUS treatment. Gadolinium-based contrast agent Omniscan was injected intravenously (20 μl/kg) 50 minutes before the MRI acquisition. Raw scans confirmed BBB opening in all 4 treated NHPs. NHPs 1 and 2 were treated at MI of 0.4 (left hemisphere) and 0.8 (right hemisphere), while NHPs 3 and 4 were treated only on the left side at MI of 0.4 and 0.8, respectively. Four representative axial slices are shown here, where BBB opening is observed as hyper-intense regions, primarily within the gray matter (white arrows). The BBB opening volume and distribution varied across the NHPs, due to differences in brain anatomy and skull structure. Scale bars: 1 cm. Abbreviations. NHP: non-human primate; FUS: focused ultrasound; MI: mechanical index.

***
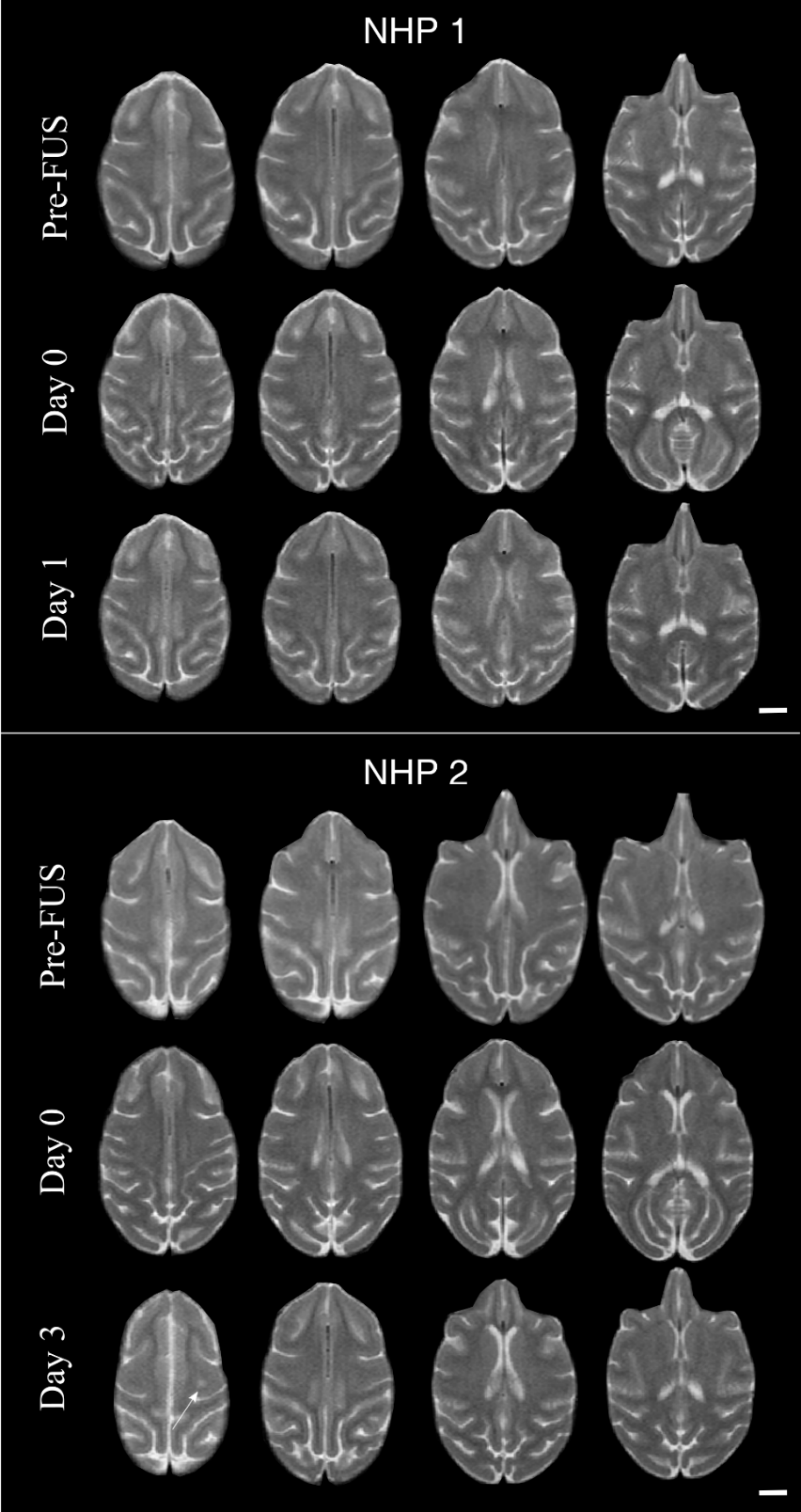
***

**Fig. S4. Axial slices of T_2_-weighted MRI scans after focused ultrasound treatment.** T_2_-weighted MRI was acquired approximately 1 hour after FUS treatment, prior to Omniscan administration and the contrast-enhanced T_1_-weighted scan. Compared to the baseline scans (pre-FUS), there was no newly-formed hyper-intense region within the treated area in either left (MI: 0.4) or right (MI: 0.8) hemispheres on days 0 and 1. Similarly there was no abnormality within the majority of the treated areas on day 3, with the exception of a 2 mm × 2 mm × 2 mm hyper-intense area on the right side (MI: 0.8) of NHP 2 (white arrow, first T_2_ slice on the left of the day 3 row). This may indicate a potential 8 mm^3^ edema, manifesting 3 days post-FUS in the vicinity of the brain-skull interface, which was visible in a single axial T_2_ slice. Scale bars: 1 cm. Abbreviations. NHP: non-human primate; FUS: focused ultrasound; MI: mechanical index.

***
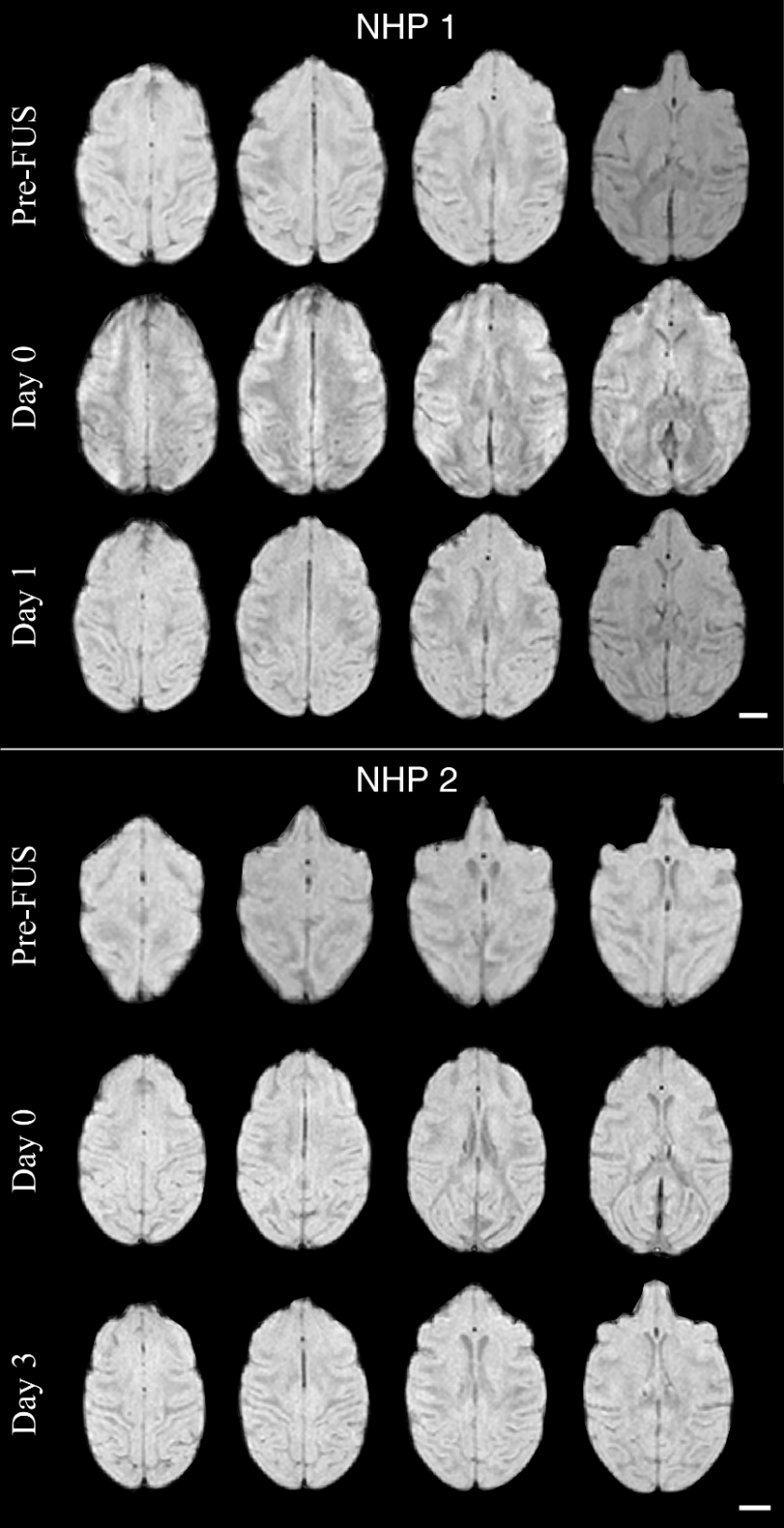
***

**Fig. S5. Axial slices of susceptibility-weighted MRI scans after focused ultrasound treatment.** Susceptibility-weighted MRI was acquired approximately 1 hour after FUS treatment, prior to Omniscan administration and the contrast-enhanced T_1_-weighted scan. Compared to the baseline scans (pre-FUS), there was no newly-formed hypo-intense region within the treated area in either left (MI: 0.4) or right (MI: 0.8) hemispheres on days 0, 1, and 3. This suggests that there was no hemorrhage due to the FUS treatment. Scale bars: 1 cm. Abbreviations. NHP: non-human primate; FUS: focused ultrasound; MI: mechanical index.

***
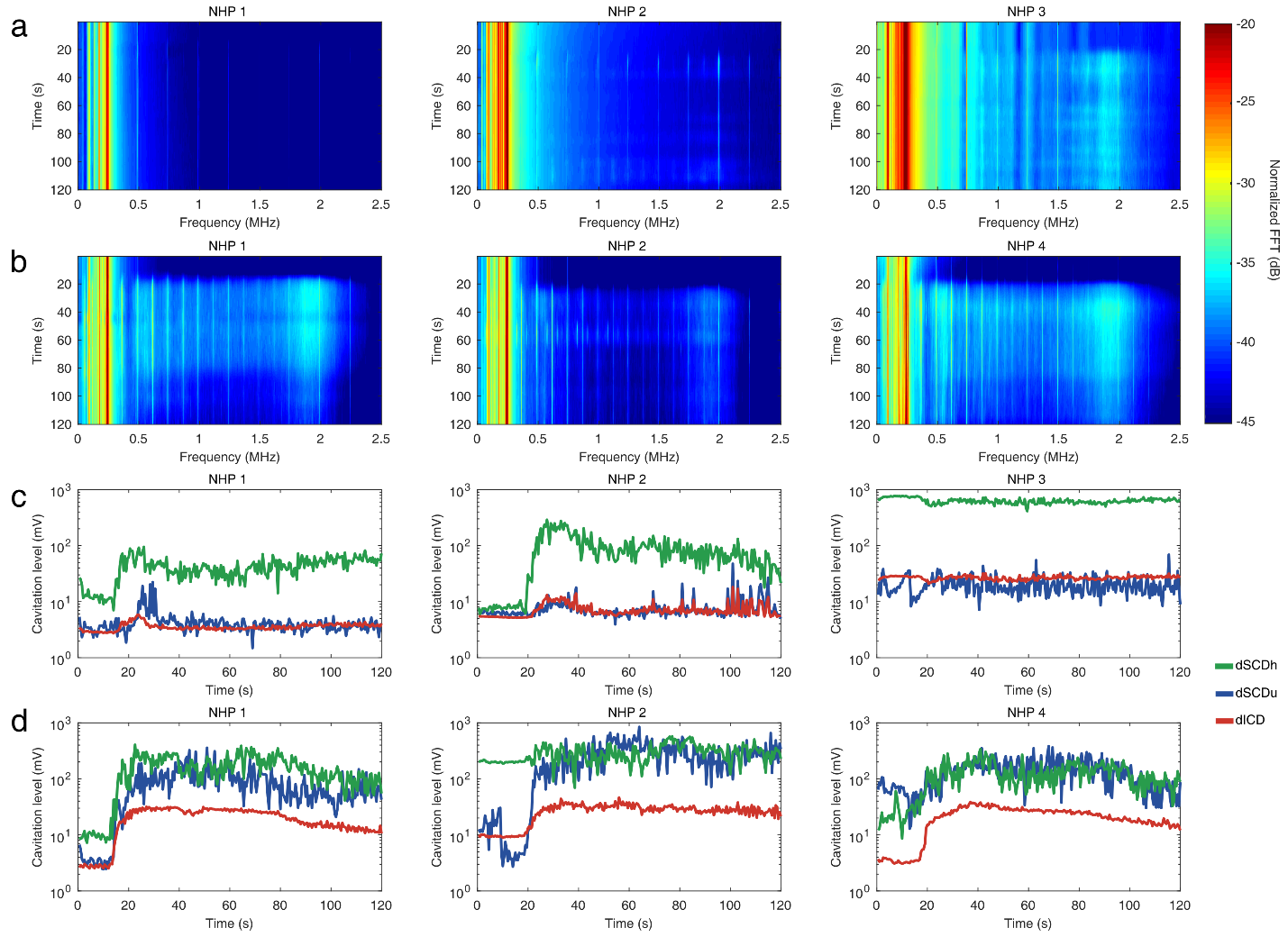
***

**Fig. S6. Spectral analysis and cavitation levels.** (**a**) Spectrograms with the frequency response of microbubbles exposed to FUS at MI of 0.4 for NHP 1 (left), 2 (middle), and 3 (right), throughout the 2-min treatment duration. Harmonic emissions dominated throughout treatment, indicating stable and harmonic oscillations of microbubbles within the vessels of the treated area. Recorded emissions had a low signal-to-noise ratio (SNR) in NHP 3 (right). (**b**) Spectrograms with the frequency response of microbubbles exposed to FUS at MI of 0.8 for NHP 1 (left), 2 (middle), and 4 (right), throughout the 2-min treatment duration. Harmonic emissions were accompanied by an increase of broadband emissions, indicating inertial collapse of the cavitation nuclei, which is related to microbubble destruction and jet formation within the blood vessels. (**c**) Evolution of harmonic stable (dSCDh, green line), ultraharmonic stable (dSCDu, blue line), and inertial (ICD, red line) cavitation levels at MI of 0.4, for NHPs 1 (left), 2 (middle), and 3 (right) throughout the 2-min treatment duration. Harmonic stable cavitation levels were at least an order of magnitude higher than ultraharmonic stable and inertial cavitation levels in NHPs 1 and 2. Recorded emissions had a low signal-to-noise ratio (SNR) in NHP 3 (right). Peaks of ultraharmonic stable cavitation coincided with peaks of inertial cavitation activity. (**d**) Evolution of harmonic stable (dSCDh, green line), ultraharmonic stable (dSCDu, blue line), and inertial (ICD, red line) cavitation levels at MI of 0.8, for NHPs 1 (left), 2 (middle), and 4 (right) throughout the 2-min treatment duration. Harmonic stable cavitation levels were on the same order of magnitude with ultraharmonic stable cavitation levels, indicating a rich mixture of volumetric and shape oscillations within the microbubble population. Inertial cavitation dose rose by up to one order of magnitude following microbubble administration, suggesting violent inertial cavitation events and microbubble collapse within the treated area. Spectrograms were acquired with a discrete FFT of the time-domain signal. Abbreviations. NHP: non-human primate; FUS: focused ultrasound; MI: mechanical index; FFT: fast Fourier transform; dB: decibel; dSCDh: stable cavitation level based on harmonic emissions; dSCDu: stable cavitation level based on ultraharmonic emissions; dICD: inertial cavitation level.

***
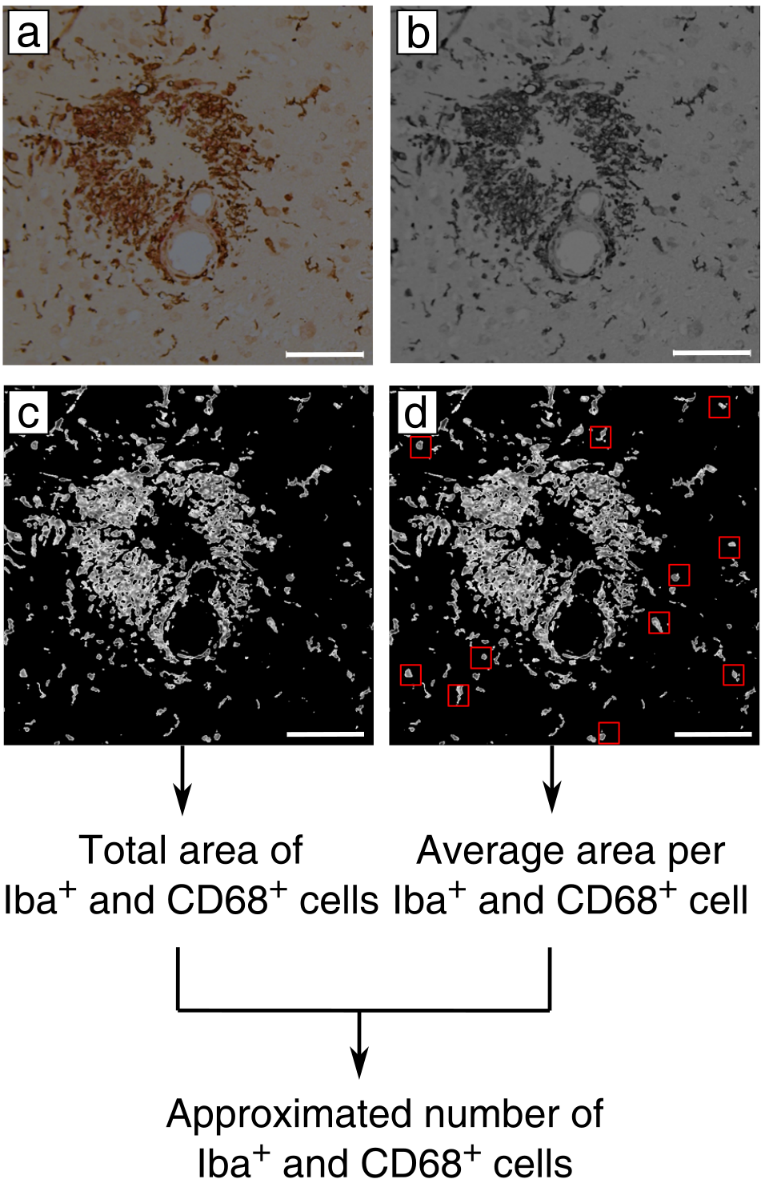
***

**Fig. S7. Iba1/CD68 quantification.** (**a**) Bright field images showing Iba1^+^ and CD68^+^ cells in perivascular areas within the treated volume were loaded in ImageJ ^16^. (**b**) RGB images were converted into 8-bit grayscale images. (**c**) The background was removed and a threshold was applied to isolate Iba1^+^ and CD68^+^ cells. The total area of microglia cells was calculated by summing all the pixels above the threshold. (**d**) A library of 10 isolated microglia without processes (red boxes) was used to calculate the average size of a single glial cell. The average Iba1^+^-CD68^+^ cell area was computed as 65 ± 33 μm^2^. To approximate the total number of cells within the image, the total area occupied by microglia was divided by the average cell area. Cell density was calculated as the number of Iba1+-CD68+ cells per mm^2^ within and out of the treatment area. This calculation was expected to overestimate cell density, since glial processes were not taken into account in the single-cell area calculation. Furthermore, this is a 2D calculation based on brain slices and may be not representative of the glial cell density in 3D brain structures. Scale bars: 200 μm. Abbreviations: Iba1: ionized calcium binding adaptor molecule 1; CD68: cluster of differentiation 68; RGB: red-green-blue image format.

***
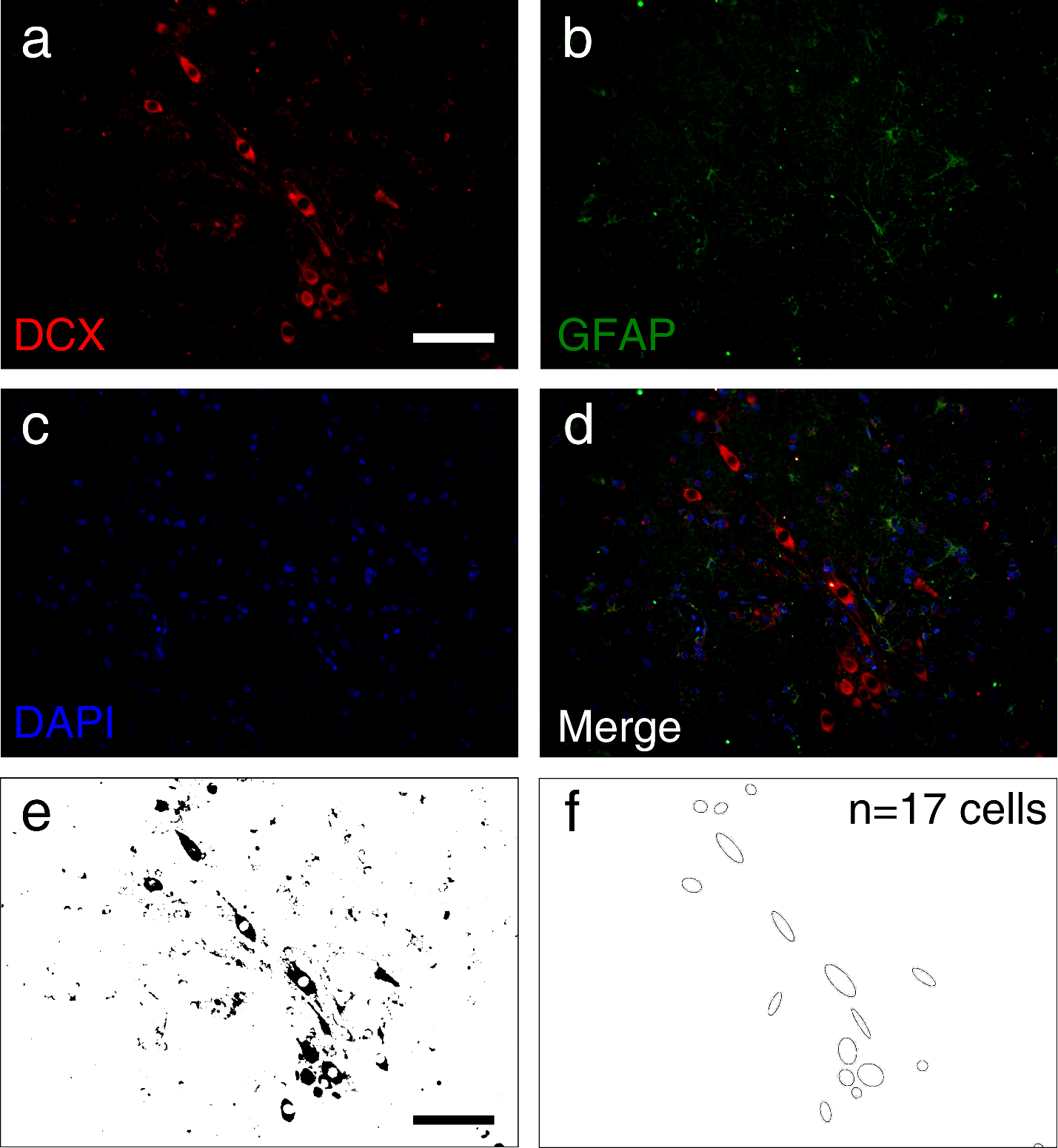
***

**Fig. S8. Doublecortin quantification.** (**a**) DCX was counter-stained with Alexa 647 fluorophore and was imaged in the red channel. (**b**) GFAP was counter-stained with Alexa 488 and was imaged in the green channel. (**c**) DAPI was imaged in the blue channel. (**d**) Channel merge. (**e**) Red channel images were used for DCX quantification. The background was removed and a threshold was applied to isolate DCX^+^ cells. (**f**) The ImageJ function “Analyze Particles” was used to count the DCX^+^ cells in each image (n = 17 cells in this example image, illustrated as ellipses). Cell density was calculated as the number of DCX^+^ cells per mm^2^ within and out of the treatment area. **DAPI did not co-localize with larger DCX^+^ cells, possibly due to the sequence of counter-staining**. Scale bars: 100 μm. Abbreviations. DCX: doublecortin; GFAP: glial fibrillary acidic protein; DAPI: 4′,6-diamidino-2-phenylindole.

***
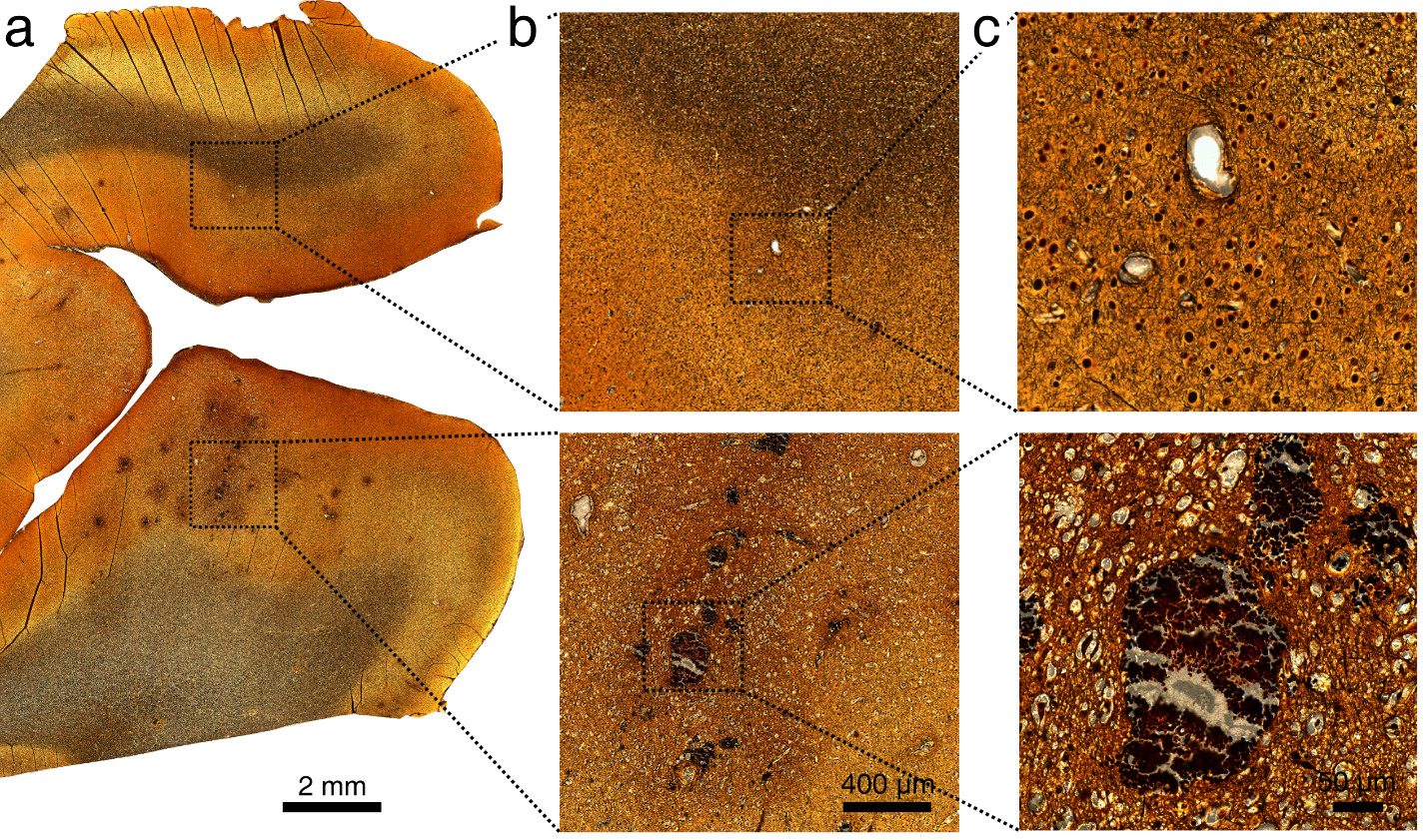
***

**Fig. S9. Axonal and neurofilament morphology after focused ultrasound treatment.** (**a**) Bielschowsky’s silver staining of the NHP 1 brain (2-day time point) revealed no axonal injury or abnormal morphology of neurofilaments within areas treated at MI of 0.4 (left hemisphere), and in most of the areas treated at MI of 0.8 (right hemisphere, top). However, there were instances of apparent mechanical damage and axonal injury in the proximity of compromised vessels (bottom). (**b**) Magnified images of the top and bottom boxes from (a). (**c**) Magnified images of top and bottom boxes from (b). Tissue damage from excessive intravascular stresses coincides with the mechanical damage observed in H&E and LFB-H&E staining (Fig. 3(b)-(c)). No abnormality was detected in the NHP 2 brain (18-day time point), in either hemisphere. Scale bars: (a) 2 mm, (b) 400 μm, and (c) 50 μm. Abbreviations. FUS: focused ultrasound; NHP: non-human primate; MI: mechanical index; H&E: hematoxylin and eosin; LFB: luxol fast blue.

***
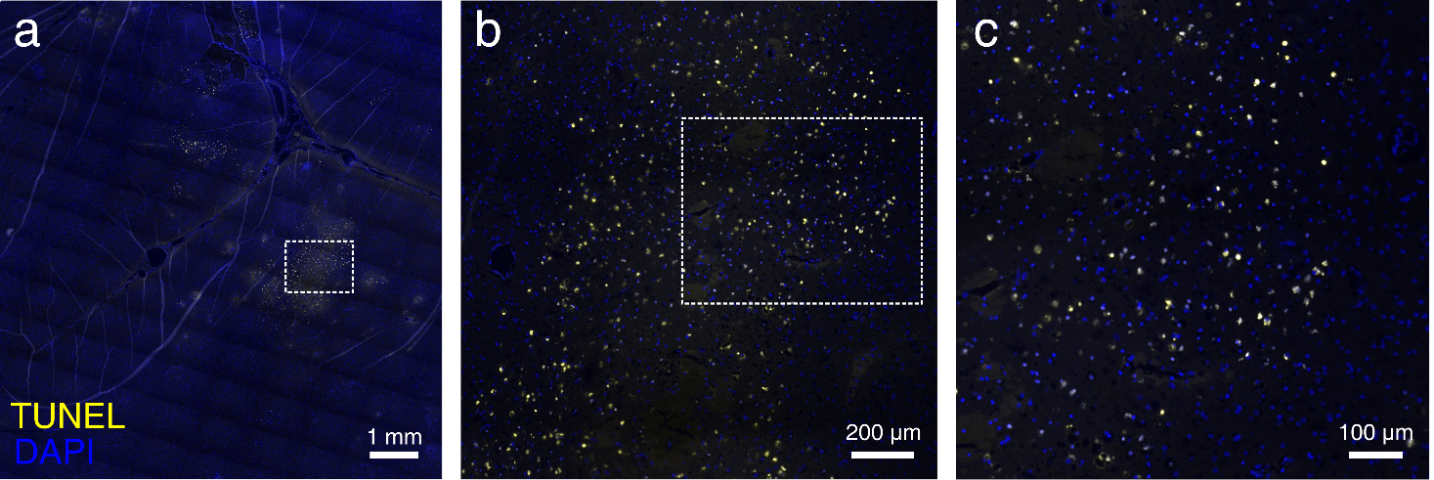
***

**Fig. S10. Focused ultrasound-triggered apoptosis.** (**a**) TUNEL assay stained cells undergoing DNA fragmentation during apoptosis within the brain area treated at MI of 0.8 (right hemisphere) in NHP 1 (2-day time point). We observed no TUNEL^+^ areas in the left hemisphere of NHP 1 (2-day time point) or in either hemisphere of NHP 2 (18-day time point). (**b**) Magnified image of the box in (a), showing a high concentration of TUNEL^+^ cells in the right hemisphere. (**c**) Magnified image of the box in (b). Scale bars: (a) 1 mm, (b) 200 μm, and (c) 100 μm. Abbreviations. TUNEL: Terminal deoxynucleotidyl transferase dUTP nick end labeling; DAPI: 4′,6-diamidino-2-phenylindole; MI: mechanical index; NHP: non-human primate.

***
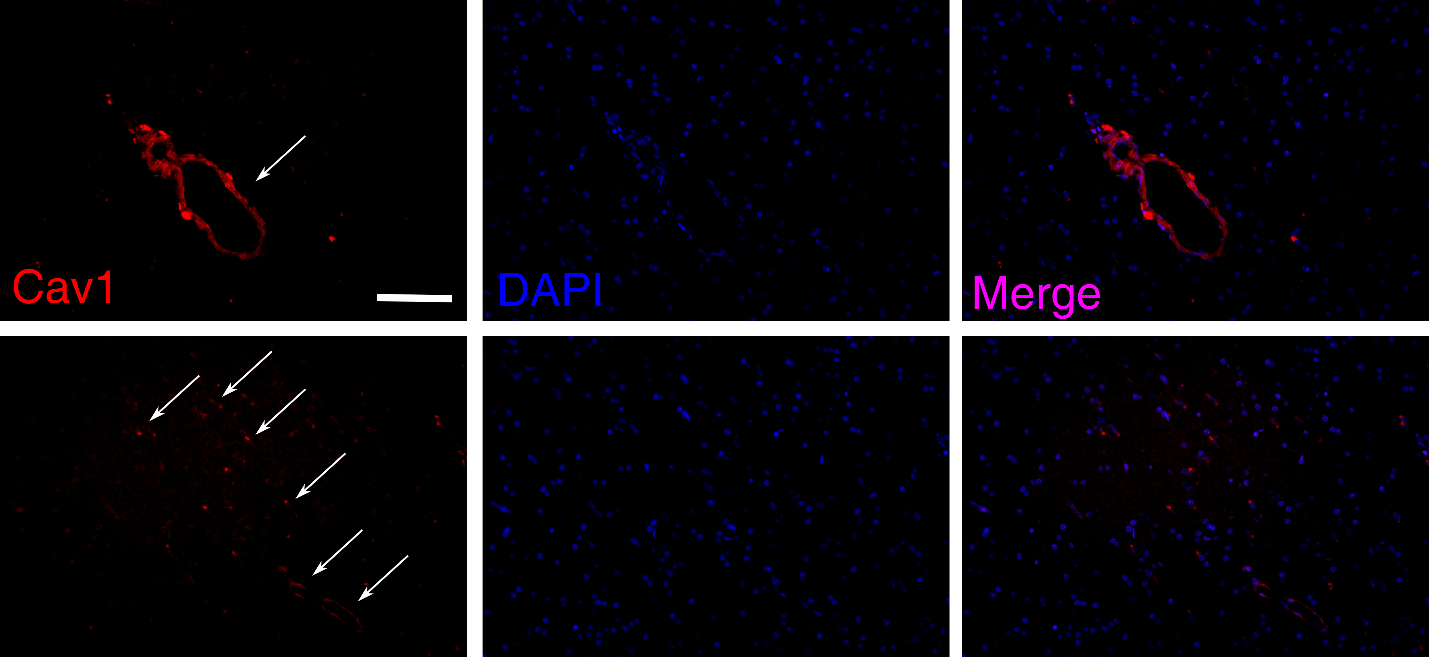
***

**Fig. S11. Caveolar endocytosis is promoted following focused ultrasound treatment.** Caveolin-mediated endocytosis is enhanced after FUS-mediated BBB opening via the upregulation of caveolin-1 expression ^20^. We detected Cav1^+^ endothelial cells in the brain area treated at MI of 0.4 (left hemisphere) in NHP 1 (2-day time point). Cav1 upregulation was more pronounced in big vessels (top), than capillaries (bottom), suggesting that enhancement of BBB permeability through the transcellular pathway is important in larger vessels. We did not detect Cav1^+^ in the brain area treated at MI of 0.8, suggesting a different BBB opening mechanism compared to the lower MI. Scale bar: 100 μm. Abbreviations. Cav1: caveolin-1; FUS: focused ultrasound; BBB: blood-brain barrier; MI: mechanical index; NHP: non-human primate.

***
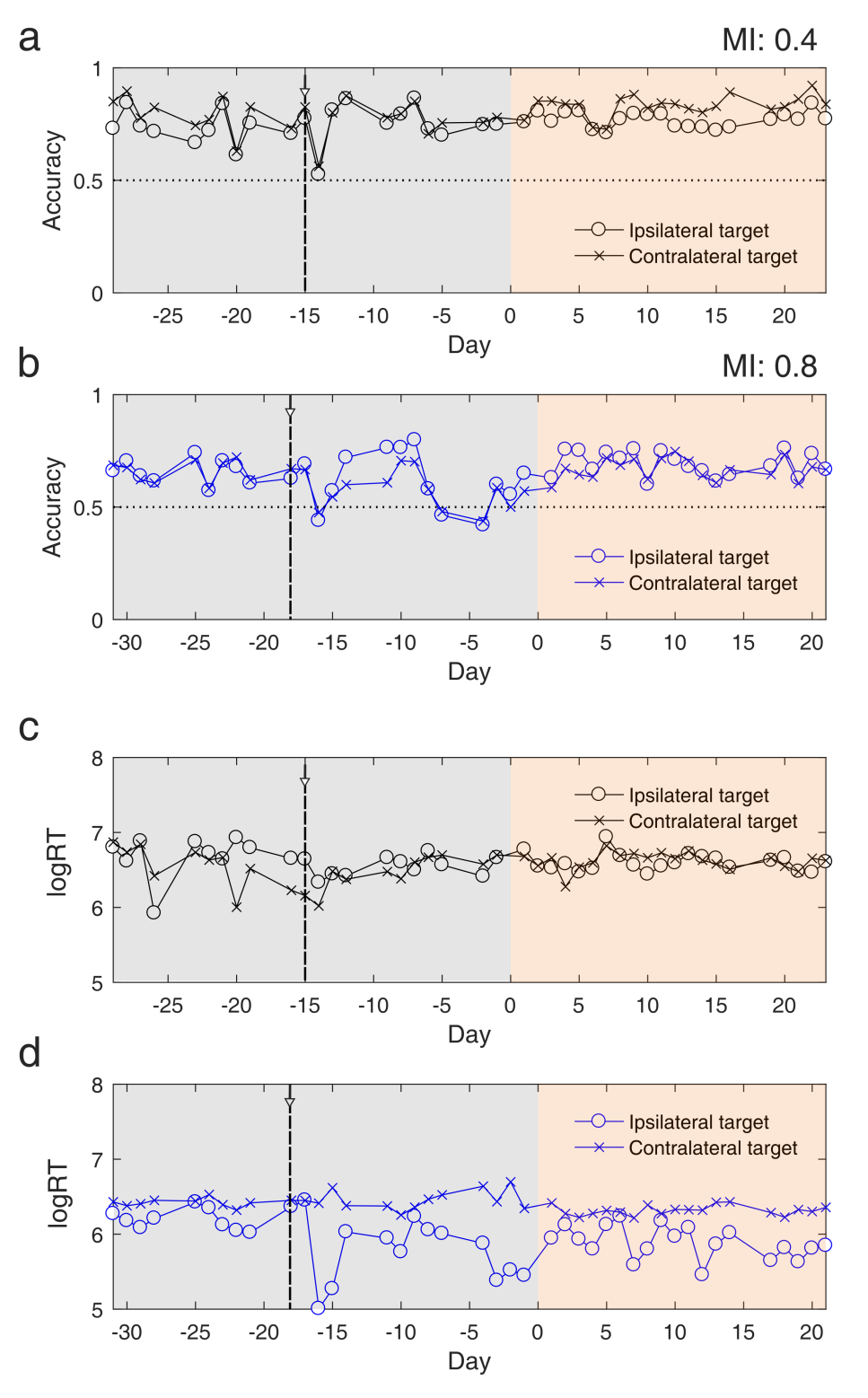
***

**Fig. S12. Accuracy and reaction time per target location.** (**a**) Accuracy during the pre-FUS (gray area) and post-FUS (orange area) periods, for ipsilateral (i.e., left, circles) and contralateral (i.e., right, crosses) targets, in NHP 3 (MI: 0.4). (**b**) Accuracy during the pre-FUS (gray area) and post-FUS (orange area) periods, for ipsilateral (i.e., left, circles) and contralateral (i.e., right, crosses) targets, in NHP 4 (MI: 0.8). (**c**) Reaction time during the pre-FUS (gray area) and post-FUS (orange area) periods, for ipsilateral (i.e., left, circles) and contralateral (i.e., right, crosses) targets, in NHP 3 (MI: 0.4). (**d**) Reaction time during the pre-FUS (gray area) and post-FUS (orange area) periods, for ipsilateral (i.e., left, circles) and contralateral (i.e., right, crosses) targets, in NHP 4 (MI: 0.8). The vertical line denotes the day of the pre-FUS MRI. Abbreviations. FUS: focused ultrasound; NHP: non-human primate; MI: mechanical index. logRT: natural logarithm of reaction time.

***
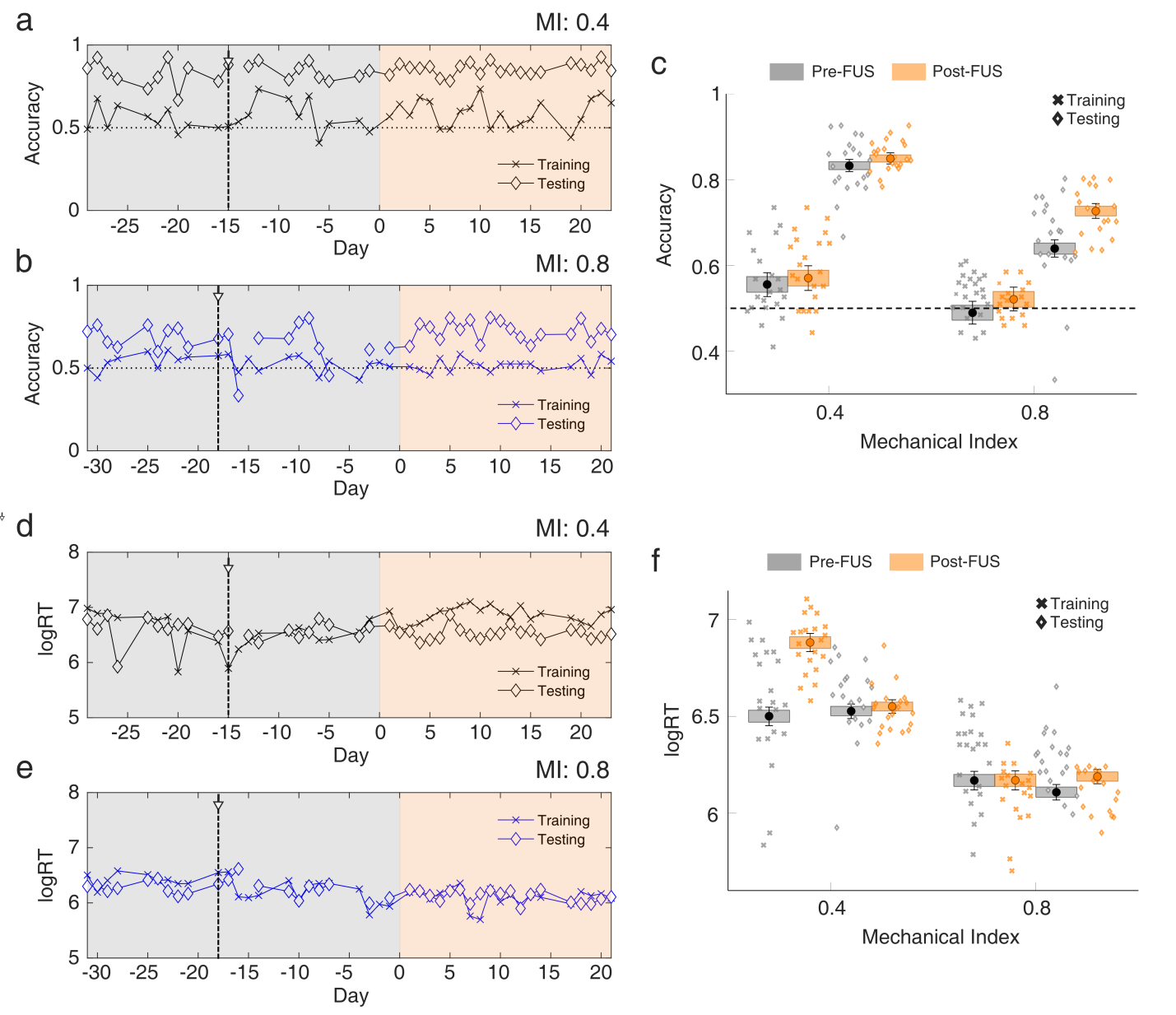
***

**Fig. S13. Accuracy and reaction time per phase.** (**a**) Accuracy during the pre-FUS (gray area) and post-FUS (orange area) periods, for training (crosses) and testing (diamonds) phases, in NHP 3 (MI: 0.4). (**b**) Accuracy during the pre-FUS (gray area) and post-FUS (orange area) periods, for training (crosses) and testing (diamonds) phases, in NHP 4 (MI: 0.8). (**c**) Accuracy intercept on day 0 during training (crosses) and testing (diamonds) phases, before (gray boxes) and after (orange boxes) FUS treatment at MI of 0.4 and 0.8. (**d**) Reaction time during the pre-FUS (gray area) and post-FUS (orange area) periods, for training (crosses) and testing (diamonds) phases, in NHP 3 (MI: 0.4). (**e**) Reaction time during the pre-FUS (gray area) and post-FUS (orange area) periods, for training (crosses) and testing (diamonds) phases, in NHP 4 (MI: 0.8). (**f**) Reaction time intercept on day 0 during training (crosses) and testing (diamonds) phases, before (gray boxes) and after (orange boxes) FUS treatment at MI of 0.4 and 0.8. Data in (c) and (f) are presented as mean ± standard deviation (n = 23 for NHP 3, i.e. MI of 0.4, and n = 21 for NHP 4, i.e. MI of 0.8). The vertical line in (a), (b), (d) and (e) denotes the day of the pre-FUS MRI. Accuracy and reaction rate for each day were the averages of all completed trials. Abbreviations. NHP: non-human primate; FUS: focused ultrasound; MI: mechanical index. logRT: natural logarithm of reaction time.

***
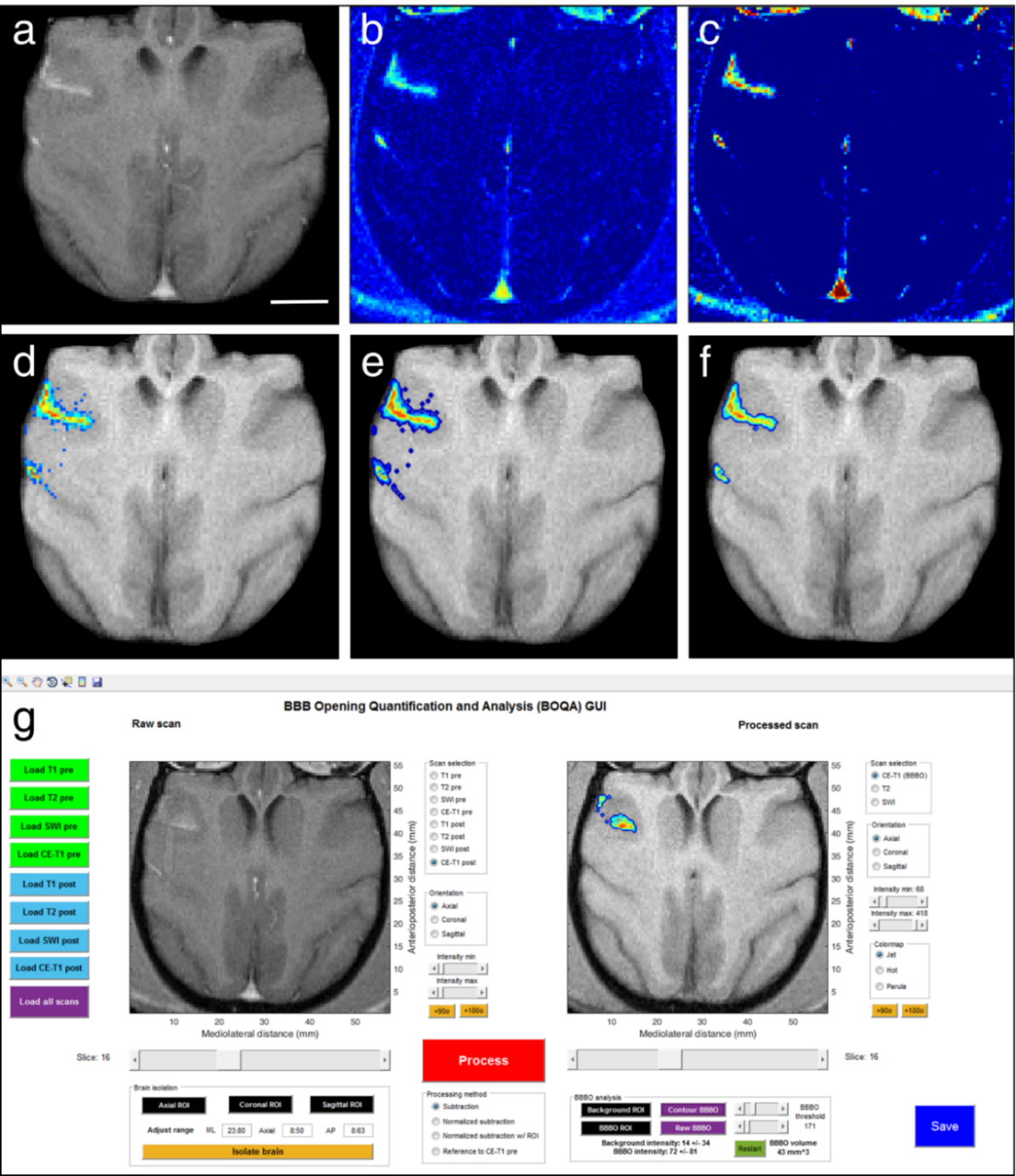
***

**Fig. S14. Blood-brain barrier opening quantification algorithm and graphics user interface.** A purpose-built graphics user interface was developed in MATLAB to quantify the BBB opening volume in NHPs treated with FUS. (**a**) The raw contrast-enhanced T_1_-weighted MRI was loaded onto the GUI. (**b**) The non-contrast-enhanced scan was subtracted from the contrast-enhanced scan to identify hyper-intense regions permeated by gadolinium. (**c**) A threshold was applied to adjust for the intensity histogram changes due to the presence of the contrast. (**d**) An ROI was selected in the treated hemisphere to isolate the region with enhanced BBB permeability, while excluding blood vessels and areas outside the brain. The resulting pixels with intensity above the specified threshold were overlaid onto the T_1_-weighted MRI scan without contrast. (**e**) Neighboring pixels with similar intensities were grouped into contour plots. (**f**) The inclusion threshold was further modified to exclude noisy pixels. The BBB opening area (in mm^2^) per slice was calculated as the area of the final contour plot. BBB opening volume per slice was calculated by multiplying the BBB opening areas by the MRI slice thickness (i.e., 1 mm). The total BBB opening volume was calculated by summing all the BBB opening volumes per MRI slice. (**g**) Screenshot of the BBB Opening Quantification and Analysis (BOQA) GUI. Raw and processed scans are shown on the left and right panel, respectively. Abbreviations. BBB: blood-brain barrier; NHP: non-human primate; FUS: focused ultrasound; GUI: graphics user interface.

**Supplementary tables**

**Table S1. Complete blood count 2 days post-treatment.**

| Test | Range | Baseline | 2 days post FUS |
| --- | --- | --- | --- |
| WBC(x10^3^/μL) | 4.7-17.4 | 4.86 | 7.86 |
| Hct(%) | 34.7-47.4 | 41.5 | 37.9 |
| RBC(x10^6^/μL) | 3.85-8.30 | 5.76 | 5.05 |
| Hgb(g/dL) | 11.0-15.6 | 13.3 | 13 |
| MCV (fL) | 52.1-103.2 | 72 | 75.1 |
| MCH | 23.4-30.9 | 23.1 | 25.7 |
| MCHC | 28.3-37.2 | 32 | 34.3 |
| RDW | 12.0-20.0 | 14.3 | 13.9 |
| RSD |  | 10.3 | 10.4 |
| Retics (K/μl) | 0.0-185.0 | 25.9 | H |
| Retics (%) | 0.0-8.75 | 0.45 | 27.34 (H) |
| Platelets(x10^3^/μL) | 259-533 | 390 | 702 (H) |

**Table S2. Complete blood count 18 days post-treatment.**

| Test | Range | Baseline | 18 days post FUS |
| --- | --- | --- | --- |
| WBC(x10^3^/μL) | 4.7-17.4 | 6.36 | 5.64 |
| Hct(%) | 34.7-47.4 | 39.4 | 47.2 |
| RBC(x10^6^/μL) | 3.85-8.30 | 5.25 | 6.4 |
| Hgb(g/dL) | 11.0-15.6 | 12.4 | 13.1 |
| MCV (fL) | 52.1-103.2 | 75.1 | 73.7 |
| MCH | 23.4-30.9 | 23.6 | 20.5 |
| MCHC | 28.3-37.2 | 31.5 | 27.8 (L) |
| RDW | 12.0-20.0 | 13.7 | 14 |
| RSD |  | 10.3 | 10.3 |
| Retics (K/μl) | 0.0-185.0 | 64.6 | 11.5 |
| Retics (%) | 0.0-8.75 | 1.23 | 0.18 |
| Platelets(x10^3^/μL) | 259-533 | 379 | 499 |

**Table S3. Cell count 2 days post-treatment.**

| **Test** | **Range(%)** | **Range** | **Baseline** | | **2 days post-FUS** | |
| --- | --- | --- | --- | --- | --- | --- |
|  |  |  | **Relative (%)** | **Absolute** | **Relative (%)** | **Absolute** |
| Basophils | 0.0-1.0 | 0-150 | 0.2 | 9.72 | 0.04 | 3.144 |
| Eosinophils | 0.0-18.7 | 0-1200 | 0.6 | 29.16 | 35.27 (H) | 2772.22 |
| Segmented | 11.6-66.1 | 2060-10600 | 44.47 | 2161.24 | 50.43 | 3963.8 |
| Lymphocytes | 17.2-80.6 | 690-4500 | 52.01 | 2527.69 | 8.88 | 697.968 |
| Monocytes | 0.0-6.7 | 0-840 | 2.71 | 131.706 | 5.38 | 422.868 |

**Table S4. Cell count 18 days post-treatment.**

| **Test** | **Range(%)** | **Range** | **Baseline** | | **18 days post-FUS** | |
| --- | --- | --- | --- | --- | --- | --- |
|  |  |  | **Relative (%)** | **Absolute** | **Relative (%)** | **Absolute** |
| Basophils | 0.0-1.0 | 0-150 | 0.04 | 2.544 | 0.09 | 5.076 |
| Eosinophils | 0.0-18.7 | 0-1200 | 12.92 | 821.712 | 1.22 | 68.808 |
| Segmented | 11.6-66.1 | 2060-10600 | 51.5 | 3275.4 | 63.63 | 3588.73 |
| Lymphocytes | 17.2-80.6 | 690-4500 | 31.68 | 2014.85 | 28.61 | 1613.6 |
| Monocytes | 0.0-6.7 | 0-840 | 3.86 | 245.496 | 6.44 | 363.216 |

**Table S5. Comprehensive chemistry panel 2 days post-treatment.**

| **Test** | **Range** | **Baseline** | **2 days post-FUS** |
| --- | --- | --- | --- |
| Total Protein | 6.0-8.0 g/dl | 6.8 | 6.9 |
| Albumin | 3.2-5.0 g/dl | 4.1 | 3.9 |
| Alk. Phos. | 57-309 u/L | 77 | 82 |
| Glucose | 34-96 mg/dl | 134 | 41 |
| Total Bilirubin | 0-1.0 mg/dl | 0.1 | 0.7 |
| Phosphorus | 3.0-6.2 mg/dl | 5 | 5.2 |
| Cholesterol | 89-228 mg/dl | 128 | 123 |
| GGT | 19-85 u/L | 47 | 47 |
| ALT(SGPT) | 0-73 u/L | 27 | 132 (H) |
| Calcium | 8.0-11.0 mg/dl | 8.8 | 8.3 |
| Creatinine | 0.4-1.0mg/dl | 1.1 | 1.2 |
| BUN | 10-26 mg/dl | 12.7 | 11.5 |
| AST(SGOT) | 2.5-48 u/L | 25 | N/A |
| Sodium | mmoI/L | 143 | 142 |
| Potassium | mmoI/L | 3.9 | 3.9 |
| Chloride | mmoI/L | 105 | 103 |

**Table S6. Comprehensive chemistry panel 18 days post-treatment.**

| **Test** | **Range** | **Baseline** | **18 days post-FUS** |
| --- | --- | --- | --- |
| Total Protein | 6.0-8.0 g/dl | 6.4 | 6.6 |
| Albumin | 3.2-5.0 g/dl | 3.9 | 4.1 |
| Alk. Phos. | 57-309 u/L | 142 | 101 |
| Glucose | 34-96 mg/dl | 106 | 63 |
| Total Bilirubin | 0-1.0 mg/dl | 0.1 | 0.2 |
| Phosphorus | 3.0-6.2 mg/dl | 5.2 | 5 |
| Cholesterol | 89-228 mg/dl | 182 | 178 |
| GGT | 19-85 u/L | 80 | 70 |
| ALT(SGPT) | 0-73 u/L | 20 | 25 |
| Calcium | 8.0-11.0 mg/dl | 9 | 9.4 |
| Creatinine | 0.4-1.0mg/dl | 1.2 | 1.2 |
| BUN | 10-26 mg/dl | 13.3 | 13.5 |
| AST(SGOT) | 2.5-48 u/L | 27 | N/A |
| Sodium | mmoI/L | 144 | 143 |
| Potassium | mmoI/L | 3.9 | 4.7 |
| Chloride | mmoI/L | 107 | 106 |

**Table S7. Neurological examination for assessment of focused ultrasound neurological safety.**

**
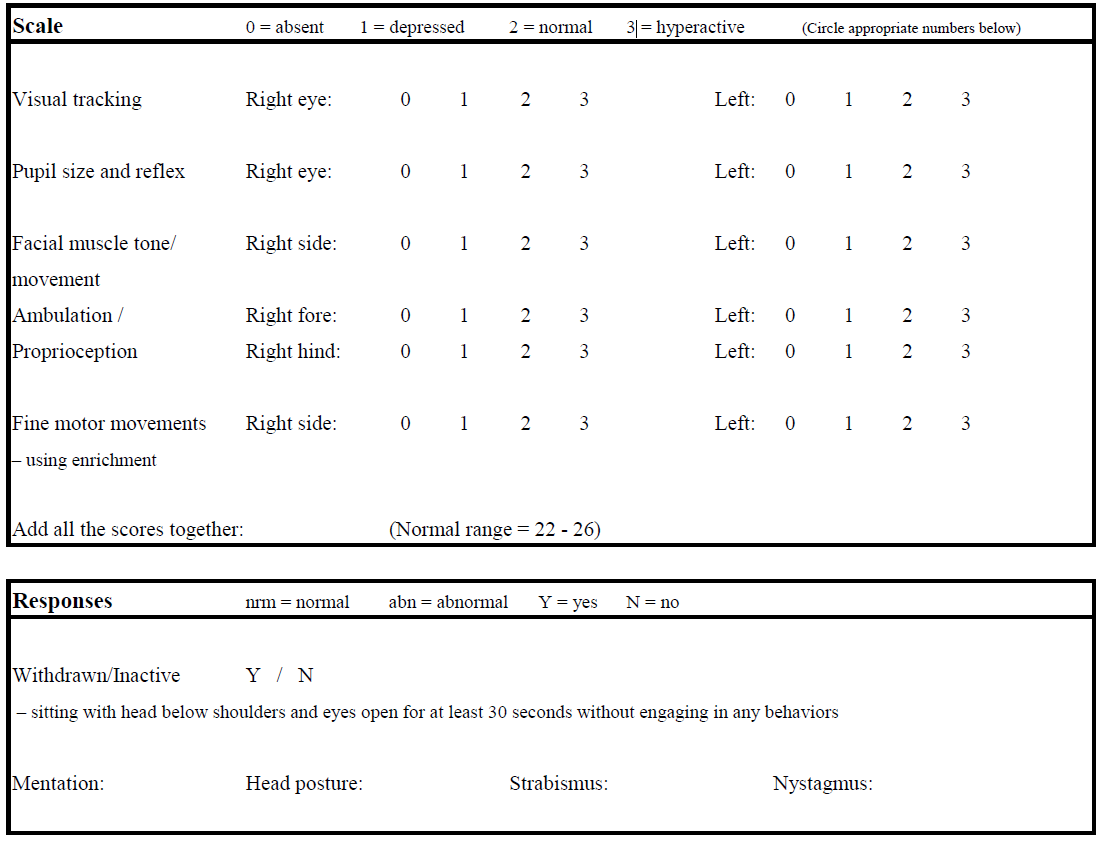
**

**Table S8. Cognitive performance overview.**

|  | Behavioral trait | MI: 0.4 | MI: 0.8 |
| --- | --- | --- | --- |
| Accuracy | Overall - Pre-FUS | 0.76 ± 0.08 | 0.62 ± 0.08 |
|  | Overall - Post-FUS | 0.8 ± 0.03 | 0.68 ± 0.04 |
|  | Left targets - Pre-FUS | 0.74 ± 0.08 | 0.63 ± 0.1 |
|  | Left targets - Post-FUS | 0.77 ± 0.03 | 0.69 ± 0.05 |
|  | Right targets - Pre-FUS | 0.78 ± 0.08 | 0.61 ± 0.08 |
|  | Right targets - Post-FUS | 0.83 ± 0.04 | 0.67 ± 0.04 |
|  | Training - Pre-FUS | 0.56 ± 0.08 | 0.53 ± 0.05 |
|  | Training - Post-FUS | 0.59 ± 0.08 | 0.52 ± 0.04 |
|  | Testing - Pre-FUS | 0.83 ± 0.06 | 0.66 ± 0.11 |
|  | Testing - Post-FUS | 0.86 ± 0.03 | 0.72 ± 0.06 |
| logRT | Overall - Pre-FUS | 6.57 ± 0.18 | 6.24 ± 0.14 |
|  | Overall - Post-FUS | 6.61 ± 0.10 | 6.12 ± 0.10 |
|  | Left targets - Pre-FUS | 6.61 ± 0.22 | 5.96 ± 0.38 |
|  | Left targets - Post-FUS | 6.60 ± 0.11 | 5.89 ± 0.21 |
|  | Right targets - Pre-FUS | 6.51 ± 0.24 | 6.44 ± 0.10 |
|  | Right targets - Post-FUS | 6.66 ± 0.11 | 6.32 ± 0.06 |
|  | Reaction time - Training - Pre-FUS | 6.56 ± 0.30 | 6.29 ± 0.21 |
|  | Reaction time - Training - Post-FUS | 6.87 ± 0.14 | 6.10 ± 0.16 |
|  | Reaction time - Testing - Pre-FUS | 6.59 ± 0.20 | 6.27 ± 0.14 |
|  | Reaction time - Testing - Post-FUS | 6.54 ± 0.11 | 6.12 ± 0.11 |

**References**

1 Pouliopoulos AN, Wu S-Y, Burgess MT, Karakatsani ME, Kamimura HAS, Konofagou EE. A Clinical System for Non Invasive Blood Brain Barrier Opening Using a Neuronavigation Guided Single Element Focused Ultrasound Transducer. *Ultrasound Med Biol* 2020; **46**: 73–89.

2 Apfel R. Sonic effervescence: A tutorial on acoustic cavitation. *J Acoust Soc Am* 1997; **101**: 1227–1237.

3 Wu S-Y, Tung Y-S, Marquet F, Downs M, Sanchez C, Chen C *et al.* Transcranial cavitation detection in primates during blood-brain barrier opening--a performance assessment study. *IEEE Trans Ultrason Ferroelectr Freq Control* 2014; **61**: 966–78.

4 Pouliopoulos AN, Bonaccorsi S, Choi JJ. Exploiting flow to control the in vitro spatiotemporal distribution of microbubble-seeded acoustic cavitation activity in ultrasound therapy. *Phys Med Biol* 2014; **59**: 6941–6957.

5 Shamout FE, Pouliopoulos AN, Lee P, Bonaccorsi S, Towhidi L, Krams R *et al.* Enhancement of Non-invasive Trans-membrane Drug Delivery Using Ultrasound and Microbubbles during Physiologically Relevant Flow. *Ultrasound Med Biol* 2015; **41**: 2435–2448.

6 Pouliopoulos AN, Choi JJ. Superharmonic microbubble Doppler effect in ultrasound therapy. *Phys Med Biol* 2016; **61**: 6154–6171.

7 Kamimura HA, Flament J, Valette J, Cafarelli A, Aron Badin R, Hantraye P *et al.* Feedback control of microbubble cavitation for ultrasound-mediated blood–brain barrier disruption in non-human primates under magnetic resonance guidance. *J Cereb Blood Flow Metab* 2018; : 0271678X1775351.

8 Gyöngy M, Coussios C-C. Passive cavitation mapping for localization and tracking of bubble dynamics. *J Acoust Soc Am* 2010; **128**: EL175–80.

9 Haworth KJ, Mast TD, Radhakrishnan K, Burgess MT, Kopechek J a., Huang S-L *et al.* Passive imaging with pulsed ultrasound insonations. *J Acoust Soc Am* 2012; **132**: 544.

10 Pouliopoulos AN, Li C, Tinguely M, Garbin V, Tang M-X, Choi JJ. Rapid short-pulse sequences enhance the spatiotemporal uniformity of acoustically driven microbubble activity during flow conditions. *J Acoust Soc Am* 2016; **140**: 2469–2480.

11 Pouliopoulos ANN, Burgess MT, Konofagou EE. Pulse inversion enhances the passive mapping of microbubble-based ultrasound therapy. *Appl Phys Lett* 2018; **113**: 044102.

12 Tung Y-S, Vlachos F, Choi JJ, Deffieux T, Selert K, Konofagou EE. In vivo transcranial cavitation threshold detection during ultrasound-induced blood-brain barrier opening in mice. *Phys Med Biol* 2010; **55**: 6141–55.

13 Pouliopoulos AN, Jimenez DA, Frank A, Robertson A, Zhang L, Kline-Schoder AR *et al.* Temporal Stability of Lipid-Shelled Microbubbles During Acoustically-Mediated Blood-Brain Barrier Opening. *Front Phys* 2020; **8**: 137.

14 Karakatsani ME, Pouliopoulos A, Liu M, Jambawalikar SR, Konofagou EE. Contrast-free detection of focused ultrasound-induced blood-brain barrier opening using diffusion tensor imaging. *IEEE Trans Biomed Eng* 2020; : 1–1.

15 Hendrickx DAE, van Eden CG, Schuurman KG, Hamann J, Huitinga I. Staining of HLA-DR, Iba1 and CD68 in human microglia reveals partially overlapping expression depending on cellular morphology and pathology. *J Neuroimmunol* 2017; **309**: 12–22.

16 Schneider CA, Rasband WS, Eliceiri KW. NIH Image to ImageJ: 25 years of image analysis. Nat. Methods. 2012; **9**: 671–675.

17 Shin J, Kong C, Lee J, Choi BY, Sim J, Koh CS *et al.* Focused ultrasound-induced blood-brain barrier opening improves adult hippocampal neurogenesis and cognitive function in a cholinergic degeneration dementia rat model. *Alzheimers Res Ther* 2019; **11**: 110.

18 Burgess A, Yeung S, Aubert I. Alzheimer Disease in a Mouse Model : MR Imaging – guided Focused Ultrasound Targeted to the Hippocampus Opens the Blood-Brain Barrier and Improves Pathologic Abnormalities and Behavior. *Radiology* 2014; **273**: 736–745.

19 Karakatsani MEM, Samiotaki GM, Downs ME, Ferrera VP, Konofagou EE. Targeting Effects on the Volume of the Focused Ultrasound-Induced Blood-Brain Barrier Opening in Nonhuman Primates in Vivo. *IEEE Trans Ultrason Ferroelectr Freq Control* 2017; **64**: 798–810.

20 Deng J, Huang Q, Wang F, Liu Y, Wang Z, Wang Z *et al.* The role of caveolin-1 in blood-brain barrier disruption induced by focused ultrasound combined with microbubbles. *J Mol Neurosci* 2012; **46**: 677–687.
